# Structural and Computational Insights into the Attenuated Innate Immune Recognition of the SARS-CoV-2 N15 Lineage, an Early-Pandemic Variant

**DOI:** 10.34133/csbj.0175

**Published:** 2026-08-03

**Authors:** Hee Chun Chung, Yoontae Jin, Sung Jae Kim, Sung Hoon Park, Hyeon Woo Chung, Su Jin Hwang, Si Hwan Ko, Van Giap Nguyen, Jae Myun Lee

**Affiliations:** ^1^ Department of Microbiology and Immunology, Institute for Immunology and Immunological Diseases, Yonsei University College of Medicine, Seoul, South Korea.; ^2^ Department of Microbiology and Immunology, Institute for Immunology and Immunological Diseases, Brain Korea 21 Project for Medical Science, Yonsei University College of Medicine, Seoul 03722, South Korea.; ^3^ Department of Companion Animal Health, Kyungbok University, Namyangju 12051, South Korea.; ^4^ Department of Veterinary Microbiology-Infectious Diseases, Faculty of Veterinary Medicine, Vietnam National University of Agriculture, Hanoi, Vietnam.

## Abstract

Functional diversification of severe acute respiratory syndrome coronavirus 2 (SARS-CoV-2) lineages influences fitness and evasion, yet early-pandemic determinants remain incompletely characterized. In this study, we investigated the molecular basis of a weakened immune phenotype of a SARS-CoV-2 isolate, N15, which shares genetic backbone with the ancestral Wuhan-Hu-1 strain, using integrated experimental observations and comprehensive computational modeling. While N15 showed replication kinetics comparable to those of MA10, Beta, and Omicron in Calu-3 cells, it induced significantly lower cytokine and interferon responses, demonstrating that efficient replication can be maintained despite attenuated innate immune activation. To identify the viral determinants driving this phenotype, we systematically evaluated the thermodynamic and structural consequences of N15-specific mutations. Structural bioinformatics analysis revealed that mutations in nonstructural protein 13 (nsp13, H290Y) and the envelope protein are (E protein, T11M) expected to have notable effects on the attenuated phenotype of the N15 strain. Specifically, the H290Y substitution in nsp13 is predicted to enhance protein stability by physically shielding a key ubiquitination site, thereby potentially promoting intracellular viral persistence and delaying host immune sensing. Furthermore, the E protein T11M substitution is predicted to reduce its channel activity via altered monomer and pentamer stability. Together, these findings suggest a mechanistic model in which the degradation-resistant nsp13 and the dysfunctional E protein ion channel serve as putative contributors to the virus’s ability to preserve replication while decreasing host innate immune responses. By generating plausible hypotheses, this work provides a structural framework and identifies specific candidate mechanisms that warrant future experimental validation to elucidate the molecular basis of attenuated viral pathogenesis.

## Highlights

• The SARS-CoV-2 N15 strain evades innate immunity with normal growth in Calu-3 cells.
• *In silico* analysis reveals the structural impacts of N15-specific mutations.
• The N15 nsp13 is predicted to be more stable than the wild-type one.
• The N15 envelope protein is predicted to have less activity than the wild-type one.

## Introduction

The emergence of severe acute respiratory syndrome coronavirus 2 (SARS-CoV-2) has led to extensive genomic diversification, with distinct constellations of mutations in nonstructural and structural proteins influencing viral fitness, tissue tropism, and immune evasion [1–3]. While much attention has been focused on variants of concern (VOCs) such as Beta and Omicron, the functional characteristics of various clinical isolates remain incompletely understood [4]. Specifically, it remains unclear whether specific amino acid substitutions can simultaneously support efficient replication in airway epithelial cells while differentially modulating pro-inflammatory and interferon responses [4].

Comparative analyses using human airway epithelial models provide valuable insights into virus–host interactions under physiologically relevant conditions. Specifically, Calu-3 cells, which express endogenous ACE2 and TMPRSS2, serve as a robust model to assess SARS-CoV-2 replication kinetics and innate immune responses, enabling the direct comparison of viral strains while minimizing confounding factors [5,6].

In this study, we investigated the biological characteristics of a SARS-CoV-2 strain designated N15 [7], in comparison with those of the MA10 [8], Beta, and Omicron variants. Phylogenetic analysis placed N15 within an early SARS-CoV-2 clade, distinct from later VOCs, allowing evaluation of its evolutionary relationship to globally circulating lineages [7]. Comprehensive genome annotation revealed that N15 harbors a limited but distinct set of nucleotide and amino acid substitutions across ORF1ab, spike, envelope, and accessory proteins relative to the Wuhan-Hu-1 reference strain [7,9].

To evaluate whether these unique genetic signatures translate into phenotypic variations, we first assessed viral replication and host immune activation in Calu-3 cells. Strikingly, despite its genetic divergence, the N15 strain exhibited replication kinetics comparable to those of MA10 and VOCs. However, N15 induced significantly lower levels of pro-inflammatory cytokines and interferons compared to the other strains. This apparent decoupling of viral replication from immune activation strongly suggests that the unique viral genetic background of N15 specifically regulates and dampens the host’s antiviral response.

To mechanistically explain the unique immune-evasive phenotype of the N15 strain, it is insufficient to rely solely on traditional virological assays to pinpoint the exact structural consequences of individual point mutations. The application of artificial intelligence (AI) and computational structural biology has emerged as an indispensable strategy for forecasting harmful viral strains utilizing mutation information [10,11]. Moreover, these computational approaches enable evaluations of thermodynamic stability and molecular flexibility that can accurately predict the phenotypic and functional consequences of SARS-CoV-2 mutations, including those in nonstructural and structural proteins [12–18]. Therefore, we employed an integrated structural bioinformatics and computational modeling approach.

By systematically applying sequence-based, structure-based, and protein-specific *in silico* analyses to the mutated proteins, we identified specific mutations in the nonstructural protein 13 (nsp13) helicase (H290Y) and the envelope (E) protein (T11M) as the candidate determinants of this immunosuppressive phenotype, suggesting possible impacts on virus–host interactions [19,20]. Given that the nsp13 helicase plays a crucial role in antagonizing host interferon signaling [21] and the pentameric E protein functions as a viroporin that modulates inflammatory pathways [22,23], capturing their structural and thermodynamic alterations generates plausible hypotheses linking specific viral mutations to the observed immune evasion. Ultimately, this study highlights how combining biological observations with advanced computational biophysics can guide future experiments to elucidate the precise molecular mechanisms underlying attenuated viral pathogenesis in the infection of early SARS-CoV-2 lineages related to the ancestral Wuhan strain.

## Materials and Methods

### Strain isolation and genome annotation

SARS-CoV-2 strains N15, MA10 (mouse-adapted strain), Beta, and Omicron were isolated from permissive cell lines under biosafety level 3 (BSL3) conditions using standard virus isolation procedures conducted at BSL3 in the Avison Biomedical Research Center, with the approval of the Institutional Biosafety Committee (IBC 2022-0320). Viral RNA was extracted from clarified culture supernatants. Complete genomes were obtained or retrieved from public databases (N15, PP195527; MA10, MT952602; Beta, OL966992; Omicron, EPI_ISL_695993) for bioinformatics analyses. Genome annotation was performed using the Wuhan-Hu-1 reference sequence (MN908947) [16] to define coding regions, mature peptides, and noncoding regions across ORF1ab, structural, and accessory genes. Nucleotide and amino acid substitutions for each strain were systematically cataloged for every annotated mature peptide. Furthermore, mutations were classified as common or rare based on Nextclade databases (https://clades.nextstrain.org/) [24], as summarized in Table 1.

### Phylogenetic analysis

To determine the evolutionary placement of the N15, MA10, Beta, and Omicron strains relative to globally circulating SARS-CoV-2 lineages, a global phylogenetic tree was inferred using the Nextclade platform [24]. The complete genome sequences of these strains were analyzed with Wuhan-Hu-1 (MN908947) [16] as the ancestral reference. Genetic distances were calculated as the number of nucleotide substitutions from Wuhan-Hu-1, and clade assignment followed the Nextstrain nomenclature. The phylogenetic positions of the SARS-CoV-2 strains were highlighted on the tree to visualize their clustering within an early SARS-CoV-2 clade and their relationship to later VOCs, providing context for the mutation patterns listed in Table 1 and Fig. S1.

### Viral growth kinetics in Calu-3 cells

The replication kinetics of the SARS-CoV-2 strains N15, MA10, Beta, and Omicron were evaluated in human Calu-3 cells. Calu-3 cell monolayers were infected with each virus at a multiplicity of infection (MOI) of 1.2 and incubated for 1 h at 37 °C to allow virus adsorption, after which the inoculum was removed, cells were washed twice with phosphate-buffered saline, and fresh maintenance medium was added. Culture supernatants were collected at predefined time points postinfection (1, 3, 6, 11, 19, 27, 36, and 50 h postinfection [hpi]), and viral RNA copy numbers were quantified by digital real-time polymerase chain reaction (dRT-PCR ) targeting the nucleocapsid (N) gene, as described in a previous study [25]. Data from 3 independent biological replicates (*n* = 3) are expressed as mean ± standard error, and differences in viral titers among strains at each time point were assessed by one-way analysis of variance (ANOVA) followed by Tukey’s post hoc multiple-comparison test using GraphPad Prism v10.5. To complement the viral RNA copy numbers, infectious virus titers (PFU/ml) were also determined by plaque assays to ensure the correlation between viral burden and infectivity.

### Cytokine and interferon gene expression analysis

Calu-3 cells were infected with the SARS-CoV-2 strains N15, MA10, Beta, and Omicron at an MOI of 1.2, and total RNA was extracted at predefined time points postinfection using the QIAamp Viral RNA Mini Kit (Qiagen, Hilden, Germany), following the manufacturer’s instructions. Viral RNA was treated with RNase-free DNase I (Enzynomics, Daejeon, Korea; Cat. No. D-1010) to remove residual genomic DNA, followed by reverse transcription using an oligo(dT)18-primed complementary DNA synthesis kit (Enzynomics; Cat. No. RT205), according to the manufacturer’s instructions. The relative expression levels of inflammatory cytokines (IL-1β, IL-6, IL-10, IL-29, and TNF-α) and interferon-related genes (IFN-α, IFN-β, ISG56, and OAS1) were quantified by RT-PCR, using the gene-specific primer sets listed in Table S1. Expression levels were normalized to a housekeeping gene (β-actin) and further normalized to mock-inoculated controls, and relative messenger RNA (mRNA) expression was calculated using the 2^−ΔΔCt^ method [26] with mock-infected cells as the calibrator. All data are presented as mean ± standard error from 3 independent biological replicates (*n* = 3), with each condition performed in technical triplicate. Statistical significance among strains and time points was evaluated by 2-way ANOVA followed by Tukey’s post hoc multiple-comparison tests to correct for multiple comparisons; distinct lowercase letters indicate significant differences between groups at the same time point (*P* < 0.05).

### *In silico* functional impact prediction of N15 mutations

**Fig. 1.**
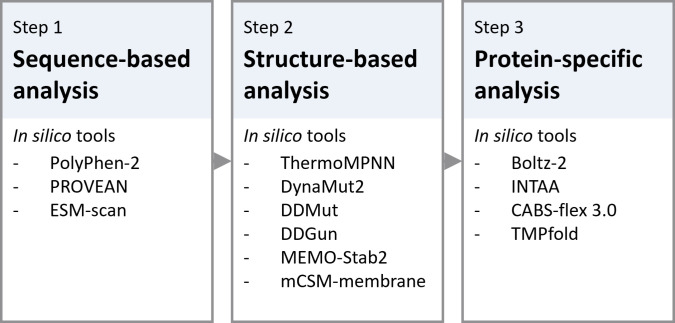
Flowchart of the *in silico* mutational analysis of proteins in the severe acute respiratory syndrome coronavirus 2 (SARS-CoV-2) N15 strain. Step 1: General mutational effects in the SARS-CoV-2 N15 proteins were predicted by sequence-based tools, using the Wuhan-Hu-1 protein sequence as a reference. Step 2: Thermodynamic changes due to the mutations were predicted by structure-based tools using protein structural data. Step 3: Protein-specific analyses were performed on selected targets based on the results from steps 1 and 2.

To systematically evaluate the structural and functional impacts of identified mutations, we introduced a multiscale *in silico* pipeline that progressively narrows from sequence-level predictions to individual protein-specific structural evaluations (Fig. 1). First, we assessed the underlying functional impairment caused by the mutations using sequence-based tools (PolyPhen-2, PROVEAN, and ESM-scan) and then calculated thermodynamic stability (∆∆G) at the structural level using consensus approaches (ThermoMPNN, DynaMut2, DDGun, DDMut, MEMO-Stab2, and mCSM-membrane). Finally, we performed customized structural analysis (Boltz-2, INTAA, CABS-flex 3.0, and TMPfold) on proteins that underwent significant changes in the preceding 2 steps.

#### Prediction of mutations on protein function

To predict the potential functional impact of amino acid substitutions identified in the SARS-CoV-2 N15 strain, *in silico* analysis was performed complementarily using the PolyPhen-2 algorithm [27], PROVEAN [28], and ESM-scan [29,30].

PolyPhen-2 (http://genetics.bwh.harvard.edu/pph2/) estimates the probability that a missense mutation is damaging by evaluating a combination of sequence-based, phylogenetic, and structural features using a naïve Bayes machine learning classifier. For each nonsynonymous mutation, the affected protein, residue change, and position (relative to the Wuhan-Hu-1 reference isolate MN908947) were entered into PolyPhen-2, and both the human divergence and human variation models were applied. PolyPhen-2 scores (0.0 to 1.0) and their corresponding qualitative predictions (benign, possibly damaging, or probably damaging), along with sensitivity and specificity values for each model, were recorded and are summarized in Table 2. Mutations were classified according to standard PolyPhen-2 categories: benign (0.0 to 0.5), possibly damaging (0.5 to 0.85), and probably damaging (0.85 to 1.0), providing an overview of the predicted structural and functional consequences of N15-specific amino acid substitutions.

PROVEAN (http://provean.jcvi.org/seq_submit.php) was utilized to predict evolutionary variation tolerance based on changes in sequence alignment scores among homologous sequences, using the Wuhan-Hu-1 reference protein sequences. A negative PROVEAN score indicates a variation that is detrimental to protein function; based on the recommended default threshold of −2.5, variations below this value were classified as deleterious, while those above thresholds were classified as neutral.

ESM-scan (https://huggingface.co/spaces/thaidaev/zsp), based on a deep learning protein language model, also utilized Wuhan-Hu-1 reference protein sequences. The ESM2_t33_650M_UR50D model [30] was selected among the supporting models considering computational speed and recency. In ESM-scan, a lower log-likelihood ratio (LLR) value is interpreted as indicating a variant unfavorable to the protein. Since ESM-scan lacks a fixed threshold, its results were interpreted using both absolute LLR scores and relative comparisons. These relative comparisons evaluated the N15 mutations against all possible variants within the protein (expressed as percentiles) and against other possible amino acid substitutions at the same position.

To establish a transparent and robust decision-making framework for prioritizing mutations, we defined a qualitative consensus criterion across these 3 distinct sequence-based algorithms. Considering that PolyPhen-2 and PROVEAN provide cutoff scores that directly evaluate the functional impact of mutations, whereas ESM-scan does not provide a cutoff value for viral protein, mutations in the N15 strain were classified as having a functional impact only if all 3 of the following conditions were met: First, PolyPhen-2 must predict the mutation as “possibly damaging” or “probably damaging” in either the human divergence (HumDiv) or the human variation (HumVar) mode. Second, the mutation must be classified as “deleterious” by PROVEAN. Third, given the limitations of ESM-scan in predicting mutational effects on viral proteins [31], we introduced “within-protein log-likelihood ratio (LLR) rank”, which calculates relative rankings as percentiles compared to all possible amino acid substitutions within the protein. This metric was applied as a relative filter, and the LLR percentile of the mutation must be below 50. Only the mutations that strictly satisfied these criteria were prioritized as primary candidates for the subsequent structural and thermodynamic evaluations.

#### Prediction of changes in structural stability due to mutation

To evaluate the effect of single amino acid substitutions on the structural stability of the SARS-CoV-2 N15 strain proteins, we predicted the change in structural thermodynamic stability (ΔΔ*G*) based on the subcellular location of the mutation. For soluble proteins such as nsp1, nsp13, and ORF8, changes in structural stability were predicted through a total of 4 *in silico*-based analyses. First, we used ThermoMPNN (https://github.com/Kuhlman-Lab/ThermoMPNN), a deep-learning-based model trained on structural and sequence patterns [32]. Second, we used DDMut (https://biosig.lab.uq.edu.au/ddmut), a Siamese network-based model that utilizes graph-based 3-dimensional local environments of mutation sites and combines convolutional layers and transformer encoders to simultaneously capture short- and long-range interactions at the atomic level [33]. Third, we used DynaMut2 (https://biosig.lab.uq.edu.au/dynamut2), a model based on normal mode analysis that considers protein dynamics and flexibility [34]. Finally, we used DDGun (https://folding.biofold.org/ddgun), which combines evolutionary information from sequence profiles with structural geometric features to calculate stability changes as a statistical energy function without a separate machine learning process [35].

For membrane proteins, various methods were applied depending on the location of the mutation. For the spike protein, since the mutated sites are located in the ectodomain, the same evaluation methods used for soluble proteins were applied [36]. For ORF3a, since residue 259 is located in the intrinsically disordered domain at the C-terminus [37], it is not resolved in all available structures of ORF3a [37,38]. Therefore, no separate predictions of stability change due to mutation were performed on the V259L substitution of ORF3a. Since the T11M mutation of E protein is located in the transmembrane domain [39,40], we used membrane-optimized *in silico* tools including MEMO-Stab2 (https://github.com/RoarBoil/MEMO-Stab2) and mCSM-membrane (https://biosig.lab.uq.edu.au/mcsm_membrane) [41,42]. MEMO-Stab2 is a machine learning model that integrates the physicochemical properties of amino acids and evolutionary information (position-specific scoring matrix), as well as environmental propensities unique to membrane proteins, such as relative position within the lipid bilayer and solvent accessibility, using a multifeature encoding method. mCSM-membrane is a model that quantifies changes in Gibbs free energy (ΔΔ*G*) by utilizing a graph-based structural feature approach and supervised learning.

To ensure consistency in result interpretation, the sign of ΔΔ*G* was standardized to indicate stabilization for negative values and destabilization for positive values. The structures of the input proteins were obtained from the Research Collaboratory for Structural Bioinformatics Protein Data Bank (RCSB PDB) [39,40,43–47], and the PDB ID information for each protein is summarized in Table 3. Furthermore, since nsp13 and the spike protein can possess multiple structural states *in vivo*, each structural state was evaluated separately.

We adopted a consensus-based framework to interpret the structural impacts of the mutations. Rather than relying on absolute ΔΔ*G* values or specific probability scores as strict quantitative thresholds of individual computational tools, we focused on the consistent directionality of the predictions across multiple tools. A mutation was considered to induce a significant structural impact only if the utilized structure-based predictors consistently indicated the same thermodynamic consequence—either overall structural stabilization (a consensus of negative ΔΔ*G* values) or destabilization (a consensus of positive ΔΔ*G* values or a high probability of destabilization calculated by MEMO-Stab2 in the case of membrane protein). This qualitative consensus approach ensures that our structural evaluation is robust against the algorithmic biases of any single tool, allowing us to prioritize mutations with high confidence for the subsequent protein-specific structural analyses.

### Additional structure-based *in silico* analysis of nsp13 and E protein variants

#### nsp13 helicase

We used Boltz-2 to predict the adenosine triphosphate (ATP)-binding affinity of wild-type nsp13 and N15 nsp13 [48]. The protein sequence and the PubChem code for ATP (5957) were provided to the model. When modeling the nsp13 and ATP-binding structure, no spatial constraints were applied to the ATP-binding site. The multisequence alignment mode used was mmseqs2_uniref_env. ATP-binding modeling for wild-type nsp13 and double-mutant nsp13 (T141I and H290Y) was performed 5 times each. For each predicted structure, the root mean square deviation (RMSD) with respect to the PDB (9I53) of nsp13 bound to ATP was measured to verify whether the predicted nsp13–ATP interaction structure was inferred to be similar to the experimental structure [45]. In Boltz-2, the predicted ATP-binding affinity is reported as a log(IC_50_) value using the IC_50_ in micromolar (μM) units.

The INTAA web server (https://bioinfo.uochb.cas.cz/INTAA/) was used to identify how the H290 residue of wild-type nsp13 and the Y290 residue of the nsp13 of N15 interact with other nsp13 residues [49]. For the input structure, the chain E of 7RDZ.pdb was used as the wild-type nsp13 structure [44]. The N15’s nsp13 structure was obtained using the mutagenesis function in PyMOL v3.1.3.1 based on a backbone-dependent rotamer library, with 7RDZ.pdb as the template [50]. Next, both wild-type and mutant structures were refined using GalaxyRefine (https://galaxy.seoklab.org/cgi-bin/submit.cgi?type=REFINE) [51]. INTAA calculated the total interaction energy (comprising both Lennard-Jones [L-J] and Coulomb potentials) profile of nsp13’s residues 141 and 290 with the CHARMM36 force field and the OBC-II environment [52].

CABS-flex 3.0 (https://lcbio.pl/cabsflex3/) was used to identify whether H290 in wild-type nsp13 and Y290 in N15’s nsp13 can block the K320 ε-NH_3_ functional group, one of the ubiquitination sites of nsp13 [53,54]. With energy-minimized structural data by GalaxyRefine, CABS-flex 3.0 was performed 7 times for each, yielding a total of 70 frames. The solvent-accessible surface area (SASA) of the K320 ε-NH_3_ functional group was calculated for all frames. Seventy SASA values per variant were pooled and used to compare the solvent accessibility of the K320 ε-NH_3_ functional group.

#### Envelope protein

TMPfold (https://opm.phar.umich.edu/tmpfold_server_cgopm) was used to calculate the association Gibbs free energy of the E protein pentamer [55]. Before using TMPfold, we set the transmembrane segment to the 8th through 38th residues and entered the structural data. The binding Gibbs free energies ($∆G_{\mathrm{asc}}$) predicted by TMPfold were tested using a paired *t* test to determine if there were significant stabilizing/destabilizing effects between the wild type and T11M.

INTAA was used to investigate microenvironmental differences caused by residue 11 substitution in the E protein. Each structure was input into INTAA to calculate the L-J potential acting on the side chain of residue 11. The Coulomb potential was excluded from this analysis because INTAA’s default implicit solvent environment is optimized for aqueous solutions and cannot accurately reflect the complex dielectric properties of a lipid bilayer, where Coulomb interactions are typically altered compared to a solution environment. Differences in the L-J potential acting on residue 11’s side chain according to E protein variants were tested using a paired *t* test, while differences in L-J potential acting on residue 11 according to conformation were tested using a *t* test. In the INTAA results, residues significantly forming L-J potentials with residue 11 were visualized by PyMOL.

Before using TMPfold and INTAA, all T11M mutant structures were derived from the closed state (PDB ID: 7K3G) and the open state (PDB ID: 8SUZ) of the wild-type E protein, which respectively contain 10 different ensembled structures derived from nuclear magnetic resonance data [39,40]. In detail, we used structures modeled with PyMOL by applying a Dunbrack backbone-dependent rotamer library to each wild-type E protein’s structural frame [50]. Both the wild-type and T11M variant’s structural data underwent an energy minimization process using the Relax application on the ROSIE web server (https://r2.graylab.jhu.edu/apps/submit/relax) [56,57]. Backbone constraints and Cartesian minimization were applied during this process. Then, all structures from the Relax application were validated by MolProbity [58].

## Results

### Global phylogenetic placement of the 4 SARS-CoV-2 strains

A global phylogenetic tree was constructed using the Nextclade platform to position the 4 SARS-CoV-2 strains used in this study, N15 (PP195527), MA10 (MT952602), Beta (OL966992), and Omicron (EPI_ISL_695993), within the context of worldwide viral diversity. Wuhan-Hu-1 (MN908947) was used as the ancestral reference, and branch positions along the *x*-axis reflect the cumulative number of nucleotide substitutions relative to this reference, whereas the *y*-axis indicates Nextstrain clade assignments. In this analysis, the N15 strain clustered within an early SARS-CoV-2 clade (19B), distinct from the later VOCs represented by the Beta and Omicron strains, which mapped to their respective VOC-associated clades. MA10 occupied an intermediate position corresponding to an early-pandemic lineage (19A), providing a bridge between ancestral-like viruses and VOCs, and together, these 4 strains span a phylogenetic spectrum from early circulating lineages (N15 and MA10) to globally dominant immune-evasive variants (Beta and Omicron), as illustrated in Fig. S1.

### Replication kinetics of the SARS-CoV-2 strains in Calu-3 cells

**Fig. 2.**
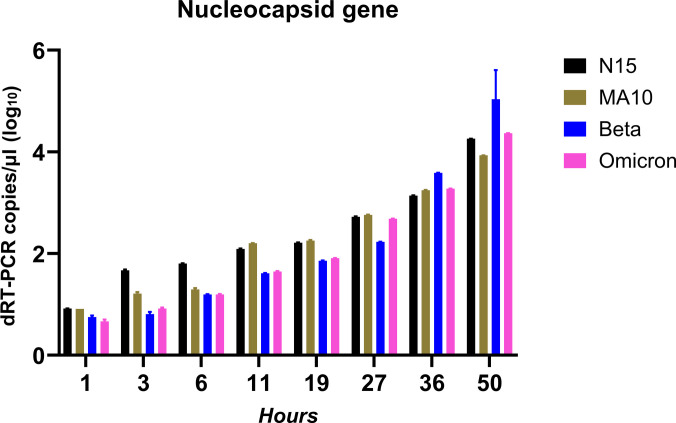
Replication kinetics of severe acute respiratory syndrome coronavirus 2 (SARS-CoV-2) strains in human Calu-3 cells. Calu-3 cells were infected with each SARS-CoV-2 strain at a 1.2 multiplicity of infection (MOI), and culture supernatants were collected at the indicated time points postinfection to determine viral titers (viral RNA copy numbers). Data are presented as mean ± standard error from independent experiments (*n* = 3, biological replicates). Differences among strains at each time point were assessed by one-way analysis of variance (ANOVA), but no statistically significant differences were detected (*P* ≥ 0.05 for all comparisons); therefore, no group-specific significance letters are indicated. All statistical analyses were performed using GraphPad Prism v10.5. dRT-PCR, digital real-time polymerase chain reaction.

Calu-3 cells were infected with the SARS-CoV-2 strains N15, MA10, Beta, and Omicron at an MOI of 1.2, and viral RNA copy numbers in culture supernatants were monitored at 1, 3, 6, 11, 19, 27, 36, and 50 hpi. During the early phase (1 to 3 hpi), viral titers remained close to baseline for all 4 strains, consistent with a period dominated by virus attachment, entry, and initial genome replication rather than productive release. From 6 to 11 hpi, viral RNA levels began to rise appreciably, and by 11 hpi, each strain showed a clear increase compared with early time points, indicating the onset of robust replication and release of progeny virions into the supernatant. At later time points (19 to 50 hpi), viral titers continued to increase and then approached peak or plateau levels, with N15, MA10, Beta, and Omicron tracing closely overlapping growth curves throughout the observation period. Across all measured time points, one-way ANOVA revealed no statistically significant differences in viral titers among the 4 strains (*P* ≥ 0.05), demonstrating that the N15 strain exhibits replication kinetics in Calu-3 cells that are broadly comparable to those of MA10, Beta, and Omicron (Fig. 2).

### Cytokine and interferon responses of the N15 strain in Calu-3 cells

**Fig. 3.**
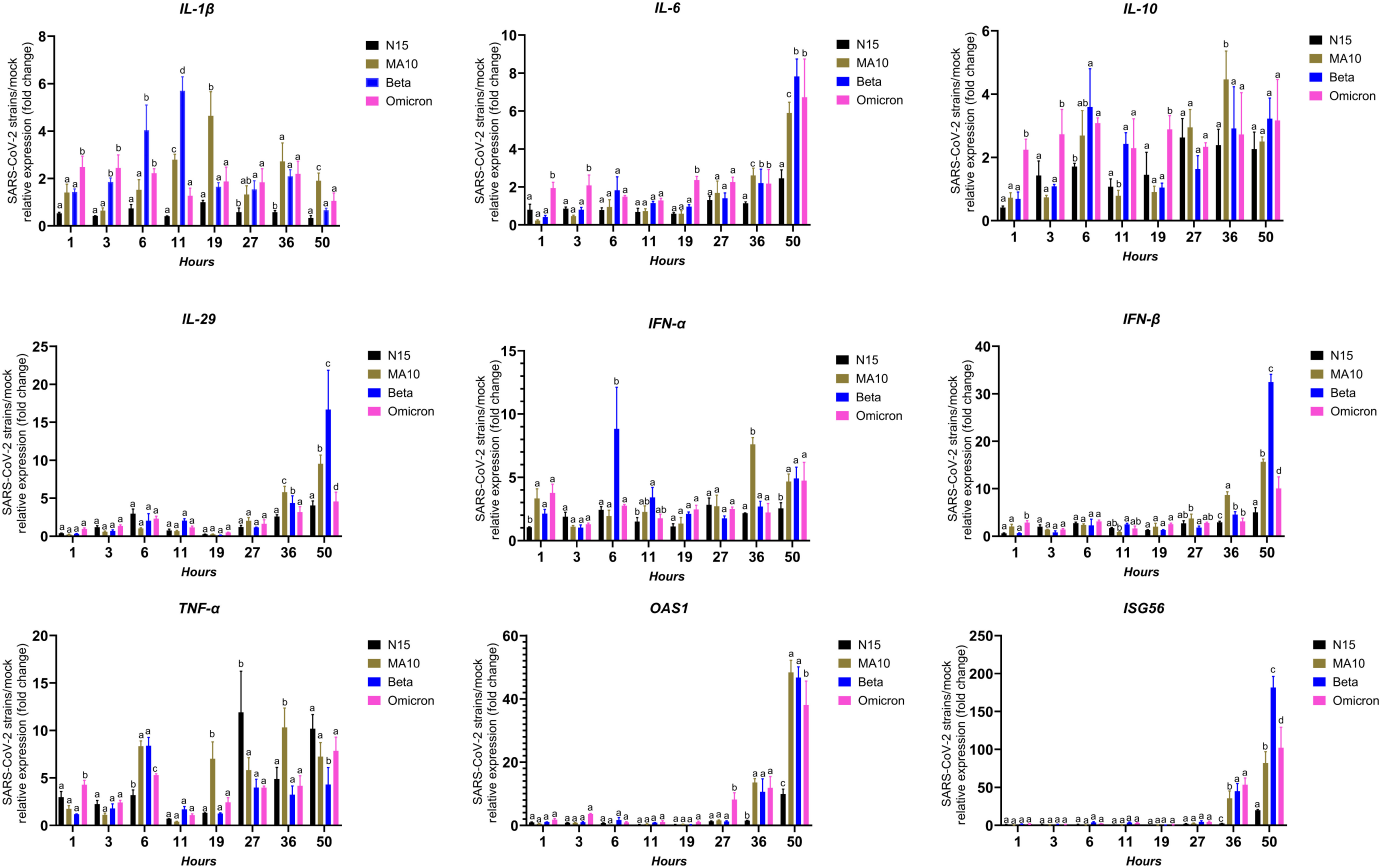
Time course of cytokine and interferon messenger RNA (mRNA) expression in Calu-3 cells infected with distinct severe acute respiratory syndrome coronavirus 2 (SARS-CoV-2) strains. Calu-3 cells were infected with the indicated SARS-CoV-2 strains, and cytokine and interferon mRNA levels were quantified by quantitative real-time polymerase chain reaction (PCR) at the indicated time points postinfection. Expression levels were normalized to a housekeeping gene and are shown relative to mock-infected controls. Data represent mean ± standard error from at least 3 independent experiments. Statistical significance was determined by 2-way analysis of variance (ANOVA) with post hoc multiple-comparison tests. Different lowercase letters (a to d) indicate statistically significant differences between groups at the same time point (*P* < 0.05).

To assess strain-specific host responses, the expression of inflammatory cytokine- and interferon-related genes was quantified over time in Calu-3 cells infected with N15, MA10, Beta, or Omicron and normalized to mock-infected controls (Fig. 3). Across a panel of inflammatory cytokines, including IL-1β, IL-6, IL-10, IL-29, and TNF-α, infection with N15 consistently resulted in lower mRNA induction compared with MA10, Beta, and Omicron at multiple time points. Notably, the induction of key pro-inflammatory mediators such as IL-6 and TNF-α was markedly attenuated in N15-infected cells. In contrast, MA10, Beta, and Omicron infections elicited robust and time-dependent up-regulation of these cytokines, reaching substantially higher peak expression levels. This pattern indicates that, despite comparable replication kinetics among the strains (Fig. 2), the N15 isolate triggers a substantially blunted pro-inflammatory response in human airway epithelial cells. A similar trend was observed for interferon-associated responses. Transcripts encoding type I interferons (IFN-α and IFN-β), as well as representative interferon-stimulated genes (ISG56 and OAS1), were induced to significantly lower levels following N15 infection than after infection with MA10, Beta, or Omicron at corresponding time points. Whereas the comparator strains generally showed progressive, time-dependent increases in interferon and ISG expression, N15-infected cells frequently remained near baseline or exhibited only modest induction. This resulted in a clear separation of expression profiles and multiple time points with significant pairwise differences.

Collectively, these findings demonstrate that the N15 strain maintains efficient replication in human airway epithelial cells while eliciting a distinctly attenuated cytokine and interferon response compared with both an early-pandemic reference strain and later VOCs. This dissociation between viral replication and host innate immune activation supports the conclusion that the unique genetic constellation of N15 is associated with qualitative alterations in virus–host interactions leading to reduced epithelial inflammatory and interferon signaling. To identify the precise molecular mechanisms and viral determinants driving this unique immune-evasive phenotype, we subsequently employed a comprehensive *in silico* structural bioinformatics pipeline to evaluate the functional consequences of N15-specific mutations.

### Genomic features and overall *in silico* mutational impacts of the N15 strain

#### Genomic features of the N15 strain

Comparative genome annotation revealed that the N15 strain harbors a distinct but limited set of nucleotide and amino acid substitutions distributed across nonstructural, structural, and accessory proteins, relative to the Wuhan-Hu-1 reference strain and in comparison with MA10, Beta, and Omicron (Table 1). In ORF1ab, N15 contained unique substitutions in nsp1 (E91A) and the helicase nsp13 (T141I and H290Y), which were not shared with the comparator strains. Additional N15-specific mutations were identified in the spike protein (N709S and E1150D), the envelope protein (T11M), ORF3a (V259L), ORF8 (L84S), and ORF10, whereas many characteristic mutations present in Beta and Omicron were absent from the N15 genome.

**Table 1.** Genome annotation of the SARS-CoV-2 strains in this study

No.	Gene/ORF	Mature peptide	Start	Stop	N15		MA10		Beta		Omicron	
					Nucleotide mutation ^a^	Amino acid mutation	Nucleotide mutation ^a^	Amino acid mutation	Nucleotide mutation ^a^	Amino acid mutation	Nucleotide mutation ^a^	Amino acid mutation
1	1ab	Leader protein (nsp1) ^b^	266	805	A537C	E91A						
		nsp2 ^b^	806	2719					C1059T, A2692T	T265I		
		nsp3 ^b^	2720	8554					C3037T, G5230T	K1665N	A2832G, C3037T, T5386G, G8393A	K856R, L2084I, A2710T
		nsp4 ^b^	8555	10054	T8767C, T8782T		C9438T	T3058I				
		3C-like proteinase (nsp5) ^b^	10055	10972					A10323G	K3353R	C10029T	T3255I
		nsp6 ^b^	10973	11842					C11750T	L3829F	C10449A, A11537G	P3395H, I3758V
		nsp7 ^b^	11843	12091			A11847G	K3861R				
		nsp8 ^b^	12092	12685			A12159G	E3965G				
		nsp9 ^b^	12686	13024								
		nsp10 ^b^	13025	13441							T13195C	
		RNA-dependent RNA polymerase (nsp12)	13442..13468	13468..16236			T15102A		C14408T	P314L	C14408T, C15240T	P314L
		Helicase (nsp13)	16237	18039	C16658T, C17104T	T1064I (=T141I ^c^) H1213Y (=H290Y ^c^)						
		3′-to-5′ exonuclease (nsp14)	18040	19620			C18060T				A18613G	I1556V
		EndoRNAse (nsp15)	19621	20658								
		2′-*O*-Ribose methyltransferase (nsp16)	20659	21552					A21137G	K2557R		
2	S	Surface glycoprotein	21563	25384	A23688G, G25012T	N709S ^d^, E1150D	C23039A, C23054T, A23056C, C23057A, C23059G	Q493K ^d^, Q498Y ^d^, P499T ^d^	C21614T, G21724T, A21801C, A22206G, G22813T, G23012A, A23063T, A23403G, C23664T	L18F, L54F, D80A, D215G, K417N, E484K, N501Y D614G A701V	C21762T, C21846T, G22578A, T22673C, C22674T, T22679C, C22686T, G22813T, T22882G, G22898A, G22992A, C22995A, A23013C, A23040G, G23048A, A23055G, A23063T, T23075C, C23202A, A23403G, C23525T, T23599G, C23604A, C23854A, G23948T, C24130A, A24424T, T24469A, C24503T, C25000T	A67V, T95I, Y145D, L212I, G339D, S371L, S373P, S375F, K417N, N440K, G446S, S477N, T478K, E484A, Q493R, G496S, Q498R, N501Y, Y505H, T547K, D614G, H655Y, N679K, P681H, N764K, D796Y, N856K, Q954H, N969K, L981F
3	3a	ORF3a protein	25393	26220	G26167T	V259L			G25563T, C25904T	Q57H, S171L	C25584T	
4	E	Envelope protein	26245	26472	C26276T	T11M ^d^			C26456T	P71L	C26270T	T9I
5	M	Membrane glycoprotein	26523	27191					C26645T		A26530G, C26577G, G26709A	D3G, Q19E, A63T
6	6	ORF6 protein	27202	27387			T27221C	F7S			A27259C	
7	7a	ORF7a protein	27394	27759								
8	7b	ORF7b	27756	27887							C27807T	
9	8	ORF8 protein	27894	28259	T28144C	L84S	T28144C	L84S				
10	N	Nucleocapsid phosphoprotein	28274	29533					C28887T	T205I	A28271T, C28311T, G28881A, G28882A G28883C, G29229A	P13L, P10S, R203K, G204R, R319H
11	10	ORF10 protein	29558	29674	G29593A							
12	Non-protein-coding region								G174T, C241T, C28253T		C241T, G29742T	

SARS-CoV-2, severe acute respiratory syndrome coronavirus 2; ORF, open reading frame; nsp1 to nsp16, nonstructural proteins 1 to 16

^a^ Nucleotide and amino acid mutations compared to the reference Wuhan-Hu-1 isolate (MN908947).

^b^ Mature peptide produced by both ORF1ab and ORF1a.

^c^ Residue numbering follows the mature nsp13 protein convention; nsp13 residues 141 and 290 correspond to positions 1064 and 1213 of the ORF1b polyprotein, respectively.

^d^ Rare mutations of SARS-CoV-2 based on the information from CoVsurver (https://gisaid.org/database-features/covsurver-mutations-app).

#### *In silico* prediction of the functional impacts of amino acid substitutions

To assess the potential functional consequences of these N15-specific amino acid substitutions, *in silico* analyses were performed using the PolyPhen-2 algorithm (applying both the HumDiv and HumVar modes), PROVEAN, and ESM-scan. The nsp1 E91A substitution yielded discordant predictions across the 3 models (Table 2). This substitution showed high PolyPhen-2 scores under both models (0.999 and 0.998, respectively) and was classified as “probably damaging” (Table S2). PROVEAN also predicted that the E91A substitution would have a deleterious effect, but its near-zero ESM-scan score and LLR rank above the 50th percentile predicted an insignificant impact on nsp1 (Tables S3 and S4). This discrepancy stems from their distinct algorithmic approaches; PolyPhen-2 and PROVEAN strongly reflect the evolutionary conservation of the residue, whereas ESM-scan, as a protein language model, evaluates mutational acceptability in the broader context of the entire protein.

**Table 2.** *In silico* predictions of the functional impacts of amino acid substitutions in the SARS-CoV-2 N15 strain using PolyPhen-2, PROVEAN, and ESM-scan. The table presents the PolyPhen-2 score, PROVEAN score, and the log-likelihood ratio (LLR) from ESM-scan for each mutation. Values are shown in bold when PolyPhen-2 predicts the mutation to be damaging or when the PROVEAN score is less than −2.5. Although ESM-scan lacks absolute criteria for classifying mutation effects, notable LLR values are shown in italics.

Gene/ORF	Mutation ^a^	PolyPhen-2 ^b^ (human divergence/variation mode) (PolyPhen-2 score)	PROVEAN ^c^ (PROVEAN score)	ESM-scan ^d^ (LLR score)
nsp1	E91A	**0.999/0.998**	**−5.000**	−0.11
nsp13	T141I	**0.838/0.444**	**−3.389**	−1.49
	H290Y	**0.997/0.994**	**−5.300**	*−4.27*
Spike	N709S	**0.963/0.745**	−0.363	*−2*
	E1150D	0.007/0.020	−0.660	−0.18
ORF3a	V259L	0.017/0.006	−0.657	−0.31
Envelope protein	T11M	**0.974/0.895**	**−3.067**	*−1.14*
ORF8	L84S	0.061/0.009	2.333	0.43

SARS-CoV-2, severe acute respiratory syndrome coronavirus 2; ORF, open reading frame

^a^ Amino acid mutations are relative to the reference Wuhan-Hu-1 isolate (MN908947).

^b^ PolyPhen-2 prediction categories: benign (0.0 to 0.5), possibly damaging (0.5 to 0.85), and probably damaging (0.85 to 1.0).

^c^ PROVEAN prediction categories: deleterious (<−2.5) and neutral (≥−2.5).

^d^ The base model used for ESM-scan is facebook/esm2_t33_650M_UR50D. No absolute categories are specified for mutation effects in ESM-scan.

The H290Y substitution in nsp13 was consistently predicted to be “probably damaging”, with scores exceeding 0.99 in both models. The T141I substitution of nsp13 was classified as “possibly damaging” and “benign” under the HumDiv mode and HumVar modes, respectively (Table 2 and Table S2). PROVEAN classified both mutations in nsp13 as deleterious (Table 2 and Table S3). ESM-scan predicted that the H290Y substitution is relatively unfavorable among all possible mutations in nsp13, reaching a consensus across other tools. However, the LLR rank of the T141I substitution was above 50th percentiles, failing to reach consensus (Table 2 and Table S4).

Analysis of structural protein mutations indicated that the spike protein substitutions N709S and E1150D failed to reach a consensus. Specifically, N709S yielded PolyPhen-2 scores of 0.963 (HumDiv mode) and 0.745 (HumVar mode), corresponding to “probably damaging” and “possibly damaging” classifications, respectively. In contrast, the E1150D substitution was consistently classified as “benign” (Table 2 and Table S2). In PROVEAN, both the N709S and E1150D variants were classified as neutral mutations (Table 2 and Table S3). ESM-scan predicted that the E1150D substitution did not have a significant impact on the spike protein, in terms of either the absolute value of LLR or a relative comparison. Although the N709S substitution had a relatively high impact on the spike protein, ranking in the top 33% among all possible mutations (Table 2 and Table S4), it failed to meet the consensus criteria due to the neutral classification by PROVEAN.

The envelope protein substitution T11M was predicted to be “possibly damaging” under both models, with high PolyPhen-2 scores and favorable specificity values (Table 2 and Table S2). PROVEAN also predicted that the T11M substitution is deleterious for the E protein (Table 2 and Table S3), and in ESM-scan, although the absolute value of LLR was small, its impact on the E protein was predicted to be in the top 29% when compared to all possible mutations (Table 2 and Table S4). Therefore, this substitution was predicted to significantly affect the E protein across all 3 tools. In addition, compared to other 19 substitutions at the 11th position, the T11M substitution is predicted to have the third most negative impact on the E protein. This distinguishes it from the mutations observed in the other N15 proteins (Table S5).

In accessory proteins, the ORF3a substitution V259L and the ORF8 substitution L84S were classified as “benign” across both PolyPhen-2 models, with low prediction scores, indicating a low likelihood of functional impact (Table 2 and Table S2). Likewise, both PROVEAN and ESM-scan determined that these variants were not deleterious (Table 2 and Tables S3 and S4). These predictions were consistent with the absence of additional high-impact mutations in these regions of the N15 genome relative to the reference strain. In summary, applying our established decision-making framework, the results indicated that the mutations in nsp13 (H290Y) and the E protein (T11M) strictly satisfied the qualitative consensus criteria across all 3 sequence-based tools, prioritizing them as primary candidates for subsequent analyses.

#### *In silico* prediction of thermodynamic stability change due to amino acid substitutions

One possible explanation for the results of quantitative RT-PCR is that mutations induce structural instability, leading to an overall decline in protein function. To verify this possibility, we evaluated changes in thermodynamic stability caused by mutations in each protein using ThermoMPNN, DDMut, DynaMut2, DDGun, MEMO-Stab2, and mCSM-membrane (Table 3), depending on the subcellular location of the mutation.

**Table 3.** Predictions of changes in protein stability due to mutations found in the SARS-CoV-2 N15 strain. *In silico* predictions were performed using ThermoMPNN, DDMut, DynaMut2, DDGun, MEMO-Stab2, and mCSM-membrane. Tools were selected based on the intracellular location of the mutations. (Negative ΔΔ*G* values indicate structural stabilization, whereas positive values indicate destabilization.) “n/a” indicates that the corresponding *in silico* tool was not applicable (see footnotes a, c, and d); for the E protein, MEMO-Stab2 (sequence-only input) and mCSM-membrane (structure input) were used interchangeably depending on input type. “-” indicates either an unresolved residue in the available PDB structures (see footnote c) or the absence of multiple, distinct conformational states in the case of nsp1, ORF3a, and ORF8.

Gene/ORF	Mature peptide	Mutation	Input	State	ThermoMPNN (ΔΔ*G*: kcal/mol)	DDMut (ΔΔ*G*: kcal/mol)	DynaMut2 (ΔΔ*G*: kcal/mol)	DDGun (ΔΔ*G*: kcal/mol)	MEMO-Stab2 (probability)	mCSM-membrane (ΔΔ*G*: kcal/mol)
1ab	Leader protein (nsp1)	E91A ^a^	9RCZ	-	0.39	0.69	−0.26	−0.1	n/a	n/a
1ab	Helicase (nsp13)	T141I ^a^	7RDZ	Apo	−0.25	0.32	0.27	−0.1	n/a	n/a
			9I53	ATP-bound	−0.44	0.05	−0.56	−0.1	n/a	n/a
		H290Y ^a^	7RDZ	Apo	−0.66	−1.93	−1.14	−1.2	n/a	n/a
			9I53	ATP-bound	−0.06	−0.10	−1.22	−1.4	n/a	n/a
S ^b^	Surface glycoprotein	N709S ^a^	6XR8	Prefusion	−0.03	0.20	−0.32	0.0	n/a	n/a
			8FDW	Postfusion	−0.31	0.11	−0.09	0.0	n/a	n/a
		E1150D ^a^	6XR8	Prefusion	0.33	0.00	1.03	0.0	n/a	n/a
			8FDW	Postfusion	−0.12	−0.13	0.94	0.0	n/a	n/a
3a	ORF3a	V259L ^c^	-	-	n/a	n/a	n/a	n/a	n/a	n/a
E ^b^	Envelope protein	T11M ^d^	7K3G	Closed	n/a	n/a	n/a	n/a	n/a	1.29
			8SUZ	Open	n/a	n/a	n/a	n/a	n/a	1.32
			Sequence	-	n/a	n/a	n/a	n/a	0.718	n/a
8	ORF8	L84S ^a^	7JTL	-	−0.11	−0.02	−0.12	0.1	n/a	n/a

SARS-CoV-2, severe acute respiratory syndrome coronavirus 2; PDB, Protein Data Bank; ORF, open reading frame; ATP, adenosine triphosphate

^a^ These mutations are located in soluble proteins or the ectodomain of the spike protein. ThermoMPNN, DDMut, DynaMut2, and DDGun were employed for stability change predictions.

^b^ Values represent the average ΔΔ*G* calculated using PDB IDs 6XR8 (prefusion form), 8FDW (postfusion form), 7K3G (closed state), and 8SUZ (open state). As the spike protein is a homotrimer, energy calculations were performed for each monomer. For the E protein, which is a homopentamer, each PDB file contains 10 stable solid-state nuclear magnetic resonance (NMR) structural models, and calculations were conducted across all 5 mutation sites.

^c^ The V259L mutation is located in an intrinsically disordered region (IDR) within the C-terminal domain of the ORF3a protein facing the cytosol. Mutations in IDRs have limited measurable effects on protein stability. Furthermore, this residue is not resolved in the available PDB structures of the ORF3a protein.

^d^ The T11M mutation in the envelope (E) protein is located in the transmembrane domain. MEMO-Stab2 and mCSM-membrane were employed for stability change predictions.

For the soluble proteins nsp1 and ORF8, the predicted ΔΔ*G* values were close to zero across all tools, and since the predictions of stabilizing or destabilizing effects varied across the tools, failing to reach clear consensus, it was predicted that changes in structural stability due to mutation would be limited. As previously established in Materials and Methods, the ORF3a V259L substitution was excluded from this prediction because no available structures of ORF3a resolve the cytosolic C-terminus tail that includes residue 259 [37,38].

Since the nsp13 helicase and the spike protein possess more than one conformation *in vivo* (ATP-bound/apo form and pre-/postfusion form, respectively), they were evaluated for each structural state. One of the nsp13 mutations, H290Y, was predicted to induce stabilization effects regardless of the structural state and the type of *in silico* tool. In detail, the predictions from DDMut, DynaMut2, and DDGun indicated profound structural stabilization for the H290Y substitution compared to other mutations found in the N15 strain. In line with the functional impact predictions, H290Y successfully satisfied our consensus criteria and was prioritized as a primary candidate based on the prediction of thermodynamic stability change. On the other hand, the T141I mutation in nsp13 failed to reach a clear consensus.

For the N709S substitution in the spike protein, the predictions of stabilizing or destabilizing effects differed among the *in silico* tools and structural states, and the predicted energy changes were within 0.5 kcal/mol. Therefore, this mutation failed to satisfy our consensus criteria and was excluded as a primary candidate. In the case of the E1150D substitution in the spike protein, although the DynaMut2 results predicted a relatively high destabilizing energy (nearly 1 kcal/mol), it was not enough to reach a clear consensus due to contradictory predictions from the other tools.

For the E protein, both MEMO-Stab2 and mCSM-membrane yielded consistent results. MEMO-Stab2 predicted that the probability of the T11M substitution causing E protein instability was 0.718. mCSM-membrane also predicted that the T11M substitution destabilizes the E protein monomer by approximately 1.3 kcal/mol, regardless of whether it was in an open or a closed conformation. Therefore, since the T11M substitution was predicted to cause consistent instability in the E protein across both *in silico* models, this mutation successfully satisfied our consensus criteria and was prioritized as a primary candidate for further analysis, similar to H290Y in nsp13.

For the soluble proteins nsp1 and ORF8, the predictions regarding stabilizing or destabilizing effects varied across the *in silico* tools, failing to reach a consensus. Consequently, these mutations were excluded from our primary candidates, consistent with the earlier sequence-based analysis. Regarding the V259L substitution in ORF3a, the mutation is located in an intrinsically disordered region, which limits the reliable prediction of thermodynamic stability. Furthermore, since this region is not resolved in any available PDB structures, the prediction was not performed.

Consequently, with the exception of the stabilizing mutations in nsp13 and the destabilizing T11M mutation in the E protein, the other substitutions were predicted to have a limited effect on the structural stability of the proteins. Therefore, we inferred from these computational predictions that the overall decrease in SARS-CoV-2 protein stability is unlikely to be the direct cause of the reduced host cell response.

### Substitutions in nsp13 and E protein are predicted to subtly interfere with their intracellular stability and activity

Since no overall decrease in the structural stability of the mutated proteins was observed, we focused on the relationship between mutations, structure, and functional biological context. Although some of our sequence-based tools predicted significant impacts for mutations such as nsp1 E91A and spike N709S, we rationally prioritized the nsp13 helicase and the E protein for subsequent structural analyses based on the consensus prediction from *in silico* models and their intracellular localization and well-established roles in cytosolic immune evasion. Specifically, the widespread reduction in cytokine and antiviral protein mRNA is typically attributed to the host shutoff function of nsp1. However, the E91A substitution in the N15 nsp1 is known to cause the loss of its host mRNA degradation activity [59], which contradicts the broad decrease in mRNAs related to host cell response. In the case of the spike protein, which plays a crucial role for SARS-CoV-2 entry, the N709S and E1150D mutations are located in the ectodomain. Thus, direct interaction with cytosolic host factors related to innate immunity is highly unlikely. Therefore, these mutations in nsp1 and the spike protein cannot fully explain the attenuated immune responses observed in our quantitative RT-PCR results. For these reasons, we directed our computational focus toward nsp13 and the E protein to mechanistically explain this unique immune-evasive phenotype.

#### The H290Y substitution of nsp13 is predicted to restrict K320 exposure and enhance stability

We first focused on the nsp13 helicase since it is a key protein capable of comprehensively inhibiting pathways related to innate immunity, such as type I interferon production [21,60–64]. Furthermore, PolyPhen-2, PROVEAN, and ESM-scan all predicted that amino acid substitutions, especially H290Y, would affect the function of nsp13. Additionally, point mutations in nsp13 were predicted to stabilize its overall structure.

**Fig. 4.**
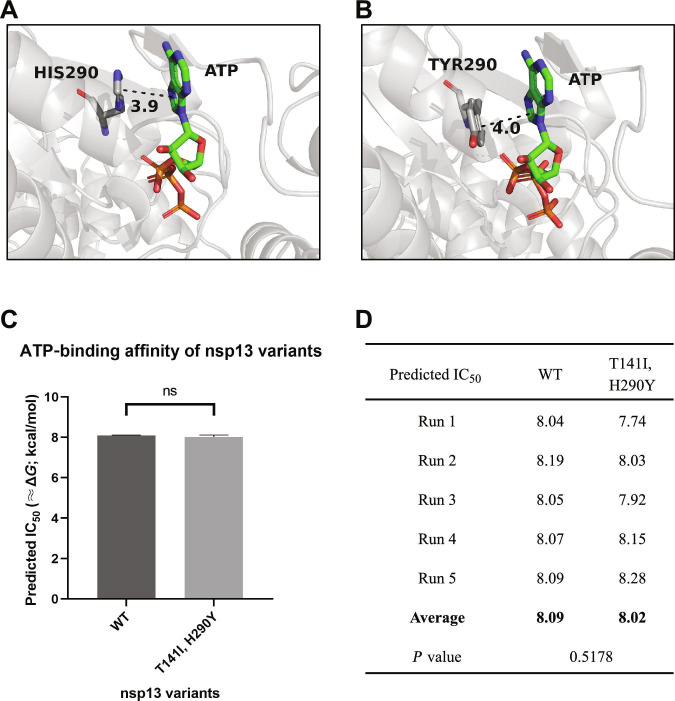
The H290Y substitution of the nonstructural protein 13 (nsp13) helicase is predicted to maintain the interaction and affinity between nsp13 and adenosine triphosphate (ATP). (A) The interaction between the H290 of wild-type nsp13 and the adenine ring of ATP predicted by Boltz-2. (B) The interaction between the Y290 of the double-mutant (T141I and H290Y) nsp13 and ATP predicted by Boltz-2. (C) Comparison of ATP-binding affinities (calculated as IC_50_, approximates Δ*G*) between the wild-type nsp13 and the N15 nsp13 predicted by Boltz-2. There is no significant change in ATP-binding affinity between the wild-type nsp13 and the double-mutant nsp13 in Boltz-2 prediction. Error bars represent the standard error of the mean (ns: *P* ≥ 0.05). (D) Raw data corresponding to panel (C), showing the predicted ATP-binding affinity of the wild-type and T141I and H290Y variant nsp13 proteins. The average of predicted IC_50_ values and corresponding *P* value (unpaired *t* test) are shown at the bottom.

Among the 2 mutated sites in nsp13, H290 is located in the ATP-binding pocket and interacts with ATP. We used Boltz-2, which can calculate ligand-binding affinity as well as perform protein structural modeling, to determine whether the H290Y substitution changes ATP-binding affinity. Boltz-2 successfully predicted the structure of ATP-bound nsp13 to be similar to 9I53 from RCSB PDB. All RMSD values of each model were below 1.0 (Fig. S2). Similar to H290 and ATP interaction, the distance between the adenine ring of ATP and Y290 was predicted to be within 5 Å, a range suitable for π–π interactions (Fig. 4A and B).

The predicted binding affinity on average for the wild type was 8.09 kcal/mol, and that for the double mutant was 8.02 kcal/mol, showing no significant difference (Fig. 4C and D). This is because both tyrosine and histidine can interact with the adenine ring of ATP via π–π stacking. This suggests that the effect of the double mutation on the ATP-binding affinity of nsp13 is limited. On the other hand, since T141 is located in the rigid stalk domain far from the functional domain, we reasoned that the direct effect of the T141I substitution on nsp13 function would be limited.

**Fig. 5.**
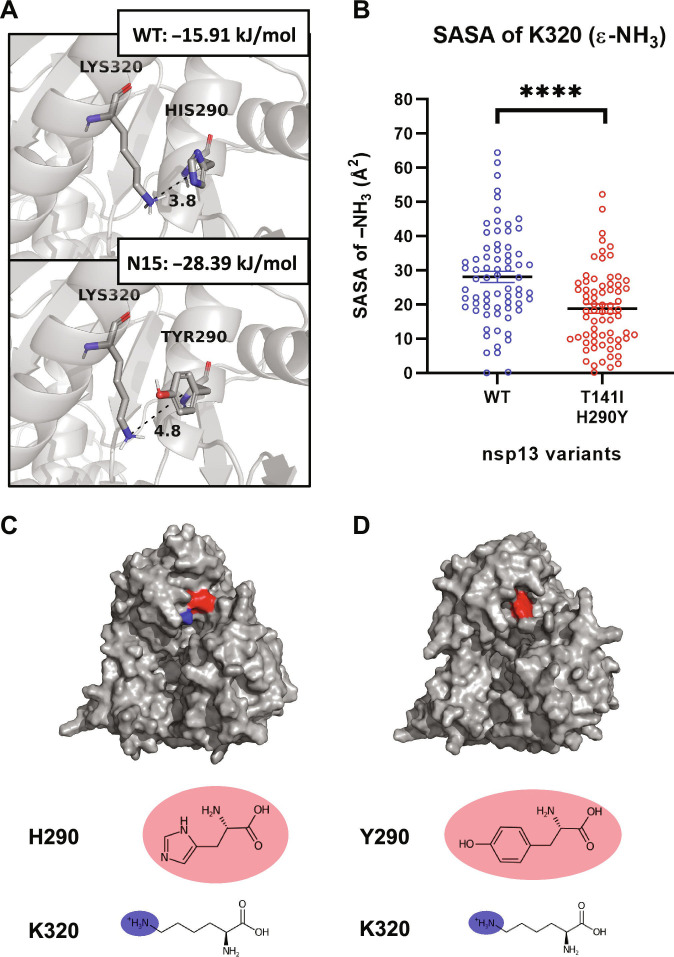
The H290Y mutation in nonstructural protein 13 (nsp13) is predicted to alter the microenvironment of the adenosine triphosphate (ATP)-binding cleft and its potential vulnerability to degradation. (A) Distance and interaction between residues 290 and K320 in wild-type (top) and N15 (bottom) nsp13. Structures were derived from Protein Data Bank (PDB) ID 7RDZ and energy-minimized using GalaxyRefine. The interaction potential between residue 290 and K320 is labeled in the upper right. (B) Solvent-accessible surface area (SASA) of the K320 ε-NH_3_ group (an nsp13 ubiquitination site) calculated via CABS-flex 3.0. Y290 reduces K320 solvent exposure more effectively than H290. Error bars represent standard error of the mean (SEM) (*n* = 7 simulations, 10 frames/trial, ****P < 0.0001). Surface exposure of the K320 ε-NH_3_ group (blue) and residue 290 (red) in (C) wild-type and (D) N15 nsp13. Animations for (C) and (D) are available in Movies S1 and S2.

INTAA was used to investigate the structural effects of the T141I and H290Y mutation on interactions with surrounding residues. The T141I substitution was predicted to enhance the interaction with L137 (from −6.06 to −21.16 kcal/mol), which is expected to increase the rigidity of the stalk domain. T141I substitution did not radically change the overall amino acid interaction profile except for L137 (Fig. S3A and B). Among the amino acids in nsp13, K320 was found to have the largest difference between the interaction with H290 and Y290 (Fig. S3C and D). In detail, while the interaction energy of H290–K320 was −15.91 kJ/mol, the interaction energy of Y290–K320 in the mutant structure was measured to be −28.39 kJ/mol, suggesting that the binding strength is potentially enhanced by approximately 1.8 times (Fig. S3E). Structural analysis revealed that the distance between residue 290 and K320 is within 5 Å, a range compatible with potential cation–π interactions (Fig. 5A). This increase in binding energy with K320 was not observed in interactions with other residues.

The INTAA result suggests that H290Y substitution can restrict the exposure of K320, one of the ubiquitination sites of nsp13. To computationally evaluate this, we analyzed the effect of H290Y substitution on the solvent accessibility of K320, the ubiquitination site of nsp13, using CABS-flex 3.0. As a result, the average SASA values of K320’s ε-NH_3_ of the wild type and N15 variant were 28.08 and 18.81 Å^2^, respectively (Fig. 5B to D and Movies S1 and S2). This suggests that Y290 effectively shields the ε-NH_3_ group of K320 compared to H290. Therefore, there is a possibility that the ubiquitination efficiency of K320 is reduced, thereby improving its stability within host cells.

#### T11M substitution of the E protein is predicted to attenuate ion channel function

Secondly, we focused on the E protein already predicted to be destabilized by T11M during the analysis of mutation effects and the prediction of changes in thermodynamic stability. The E protein is involved in NLRP3 activation, triggering IL-1β and IL-18 secretion that can propagate inflammatory signals to neighboring cells.

The *in silico* tools that we introduced so far have their own limitations in evaluating the stability change and characteristic change due to the T11M substitution in the pentameric state of the E protein. First, the tools for predicting the effects of mutations on proteins are sequence-based, not structure-based, predictions. Second, thermodynamic stability change prediction tools are used to assess the stability of monomers or subunits, but their accuracy in assessing the stability of oligomeric proteins is lower compared to that for monomers [65].

**Fig. 6.**
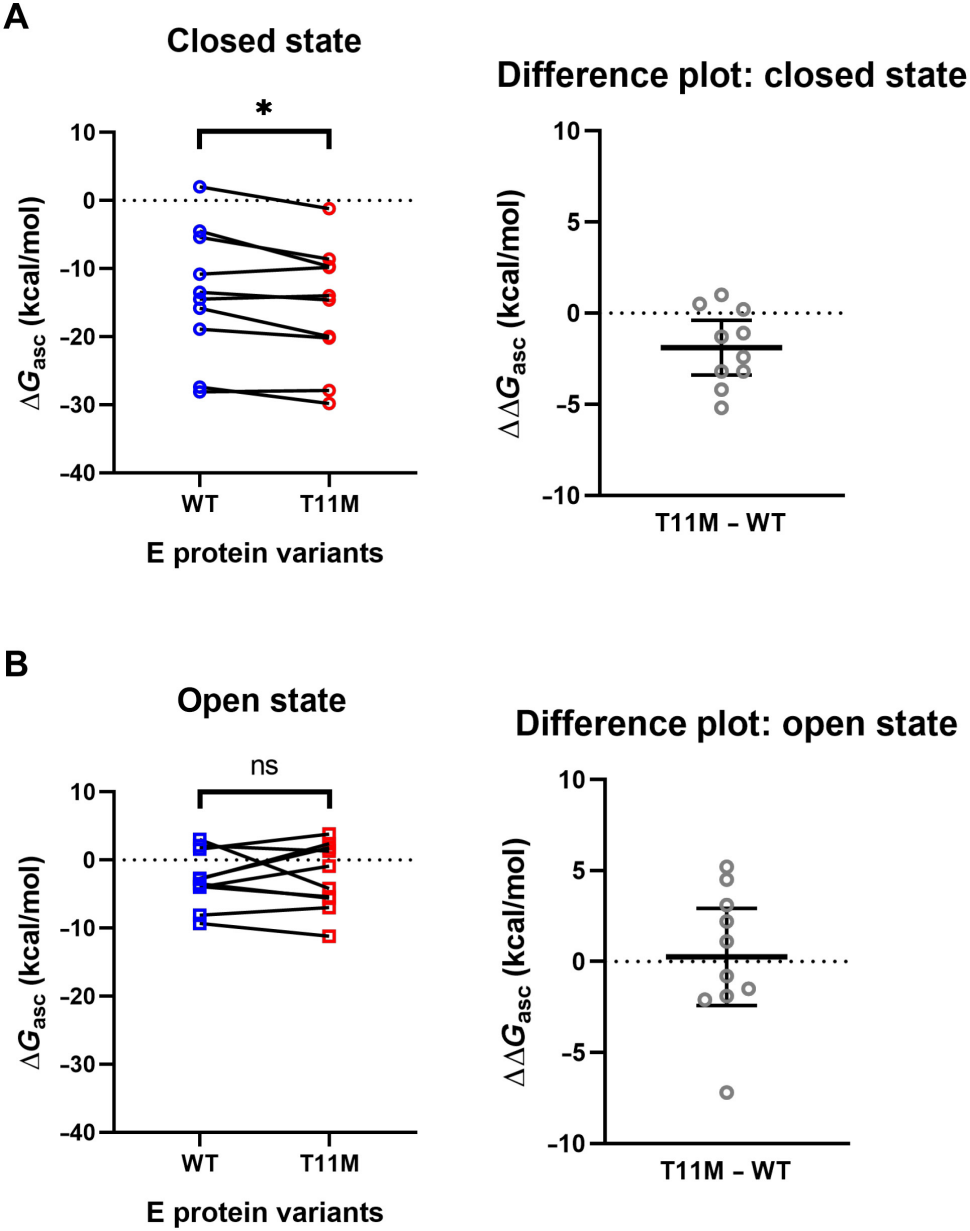
Association Gibbs free energy (kcal/mol) calculated by TMPfold. Graphs in the left panels represent the individual $∆G_{\mathrm{asc}}$ of each structural frame of the E protein. Graphs in the right panels represent $∆∆G_{\mathrm{asc}}$. A negative value indicates that the T11M mutant is more stable than the wild-type E protein. (A) The association energy difference in the closed state. The difference plot generated by a paired *t* test is illustrated in the right panel. (B) The association energy difference in the open state. The difference plot generated by a paired *t* test is illustrated in the right panel (*P < 0.05; ns, *P* ≥ 0.05).

To overcome this limitation, we used TMPfold designed to evaluate the stability of alpha helices in transmembrane domain. Before we used TMPfold, all input structural data of the E protein were refined by the Relax application in the ROSIE web server and then validated by MolProbity (Table S6). TMPfold analysis showed that the T11M substitution stabilized the closed state of the E protein to −1.89 kcal/mol based on the average of $∆∆G_{\mathrm{asc}}$. In the open state, the average of $∆∆G_{\mathrm{asc}}$ was calculated to be +0.26 kcal/mol, which was statistically insignificant (Fig. 6).

**Fig. 7.**
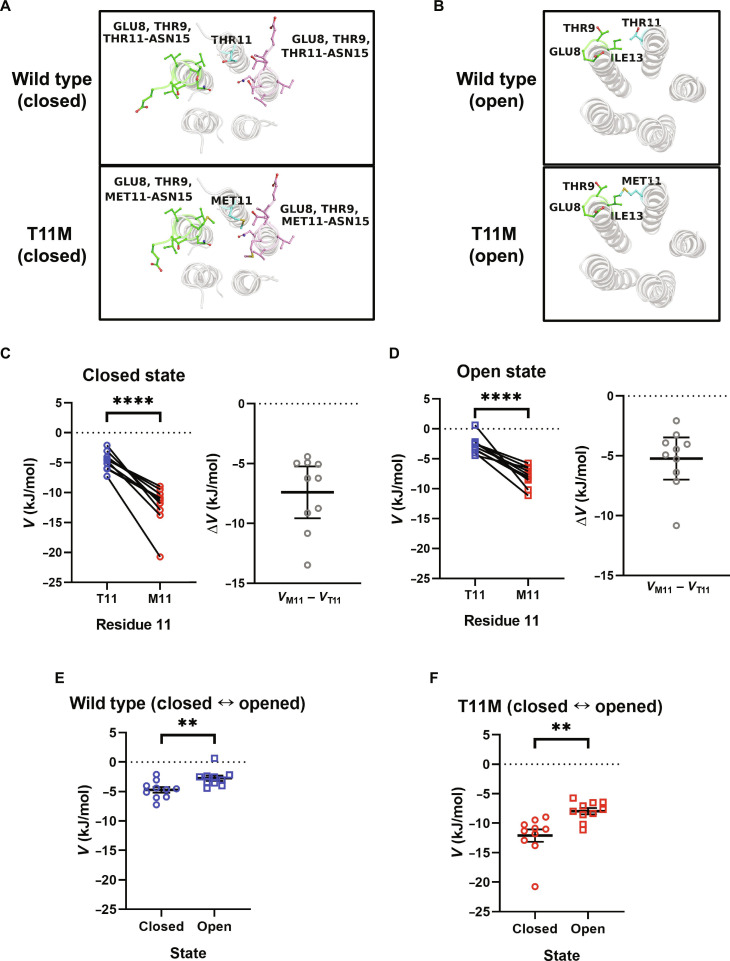
Lennard-Jones (L-J) potentials acting on residue 11 with other residues in the adjacent chains of the E protein, calculated by INTAA. Interactions between residue 11 and other residues in the adjacent chain of the (A) closed and (B) open E protein for each variant. Structures were visualized using PyMOL; residues appearing distant from residue 11 but interacting in other structural frames are also labeled. Comparison of the total L-J potential acting on residue 11 in the (C) closed and (D) open states (left panels), with difference plots generated by paired *t* tests (right panels). Comparison of the total L-J potential acting on (E) T11 and (F) M11 between the closed and open states. Error bars represent the 95% confidence interval for the difference plots in (C) and (D) and the standard error of the mean in (E) and (F). The “*V*” on the *y*-axis represents the L-J potential (**P < 0.01; ****P < 0.0001).

To investigate the microenvironmental differences caused by the residue 11 substitution, INTAA was utilized to identify which residues in the adjacent chains of the E protein interact with residue 11 (Fig. 7A and B). In the closed state, residue 11 interacts with the N-terminus of the adjacent transmembrane domain, specifically including E8, T9, L12, I13, V14, and N15, and its counterpart (residue 11). In contrast, only E8, T9, and I13 interact with residue 11 in the open state. We then calculated the L-J potential using INTAA to determine the energy changes between residue 11 and these specific interacting residues, excluding Coulomb potentials to avoid electrostatic artifacts in the membrane environment. Among these, L12 and the counterpart residue 11 form stronger interactions with M11 than with T11 in the closed state. In the open state, T9 and I13 exhibit similarly enhanced interactions with M11 (Fig. S4).

Based on these enhanced specific interactions, we quantified the total L-J potentials acting on residue 11 in the wild-type and T11M pentamers. As expected, residue 11 experienced a larger attractive total L-J potential in both the closed and open states (Fig. 7C and D). The total L-J potential acting on the 11th residue in the closed state was on average about −7.40 kJ/mol lower than that of the wild-type pentamer. In the open state, this value was −5.23 kJ/mol lower. Comparing the 2 states, the L-J potential difference between the open and closed conformations was larger for the T11M pentamer than for the wild-type pentamer (Fig. 7E and F). In detail, the L-J potential difference between the closed and open states of the wild-type E protein was about 1.97 kJ/mol on average, while for the T11M pentamer, it was about 4.15 kJ/mol. Therefore, the stabilization of the pentameric state due to the T11M substitution can reduce the conformational change of the channel, which is essential to its ion channel activity.

## Discussion

The present study demonstrates that the SARS-CoV-2 isolate N15 exhibits replication kinetics in human Calu-3 airway epithelial cells that are indistinguishable from those of the MA10, Beta, and Omicron strains (Fig. 2). This finding indicates that the distinct genetic constellation of N15 does not compromise its basic replicative fitness in human airway epithelium. Importantly, this observation rules out reduced viral replication as a confounding factor and supports the conclusion that the attenuated cytokine and interferon responses elicited by N15 reflect qualitative differences in virus–host interactions rather than differences in viral burden (Fig. 3) [3,6]. In this study, this is important because it implies that the attenuated inflammatory and interferon responses associated with N15 are unlikely to be secondary to reduced viral burden but rather reflect qualitative differences in virus–host interactions [6].

To systematically identify the viral determinants driving this unique immune-evasive phenotype, we evaluated the structural and functional consequences of N15-specific mutations using a comprehensive computational pipeline. Recognizing that *in silico* predictions can vary based on the underlying algorithms, we adopted a consensus-based approach that cross-validated results from multiple tools operating on distinct algorithmic principles. This conservative selection criterion allowed us to minimize potential false positives and focus our mechanistic interpretations on mutations where both functional impact and thermodynamic stability were consistently altered, providing a potential link between genetic variation and the observed host response.

PolyPhen-2, PROVEAN, and ESM-scan, which are *in silico* tools based on distinct algorithmic principles, were employed to predict the functional impact of each mutation. Applying our qualitative consensus criteria across these 3 distinct tools, only the H290Y substitution in nsp13 and the T11M substitution in the E protein consistently met all conditions for significant functional impact (Table 2 and Tables S2 to S6). These predictions align with the functional roles of these residues—specifically, H290 in nsp13 for ATP binding and T11 in the E protein as an essential component of the polar network required for calcium ion conduction. Although individual predictors assigned high damage scores to variants possessing distinct functional features, such as nsp1 E91A (involved in host mRNA degradation) and spike N709S (a glycosylation site), these substitutions exhibited discordant predictions across the algorithms and failed to reach consensus. While strict consensus filtering can potentially omit variants highlighted by single tools, this conservative threshold was intentionally adopted to minimize false positives, ensuring that only candidates with robust, multi-algorithmic support were prioritized for subsequent structural and biological evaluations.

Notably, despite being a tool trained on population genetic diversity data targeting only human proteins, PolyPhen-2 demonstrated prediction results that were highly consistent with those of the other tools. Furthermore, beyond this study, it has been used as a tool to predict the impact of mutations on SARS-CoV-2 [12,66,67]. This suggests that these residues are evolutionarily strongly conserved and that their functional constraints may be partially shared between host and viral proteins. For ESM-scan, the lack of a well-defined threshold made it possible to assess the functional impact of the N15 mutation only through relative comparison with all possible point mutations within the protein. Nevertheless, evaluating the impact of mutations using the relative comparison method based on ESM-scan was feasible.

We evaluated not only general mutational effects but also the thermodynamic stability of the proteins to determine whether there was an overall decline in protein function (Table 3). Similar to the prediction of mutation effects, we utilized multiple *in silico* tools established in different ways to predict the thermodynamic stability caused by mutations. Most mutations did not appear to have a significant effect on the proteins. This was determined by the inconsistent signs of the predicted values across the *in silico* tools. However, for the H290Y substitution in nsp13, a consistent stabilization pattern was observed across the models, whereas a significant destabilizing effect was predicted for the T11M substitution in the E protein. Therefore, we prioritized the H290Y substitution in nsp13 and the T11M substitution in the E protein among all mutations found in the SARS-CoV-2 N15 strain. Furthermore, both nsp13 and the E protein are directly associated with the suppression of the host immune response.

Among the mutations with the greatest predicted impact, nsp13 mutations are particularly noteworthy. Nsp13 plays a crucial role in the viral RNA unwinding and replication complex, and previous studies have shown that nsp13 can inhibit host factors such as IRF3, TBK1, and STAT1, thereby disrupting type I interferon production, as well as the JAK–STAT and NF-κB pathways [21,60–64,68]. Due to these characteristics, the nsp13 in the N15 strain is considered an important factor that enables the maintenance of low levels of mRNA encoding various cytokines and antivirals during N15 infection.

Using INTAA, we confirmed that mutant nsp13’s Y290 interacts more strongly with K320 than H290 in wild-type nsp13 (Fig. S3). This result is consistent with predictions regarding changes in structural stability due to mutations. The significant energy stabilization observed from ThermoMPNN, DDMut, DynaMut2, and DDGun may be attributed to Y290–K320 interaction.

Given the assumption that ubiquitin conjugation requires the target lysine to be sterically accessible, we investigated whether the H290Y substitution alters the surface exposure of K320. This is based on a recent multi-omics study that mapped E3 ligases (BRAP, RNF25, and TRIM27) interacting with nsp13 during active SARS-CoV-2 infection in Calu-3 lung epithelial cells and simultaneously confirmed that K320 is the ubiquitination site [54]. Using CABS-flex 3.0, we observed that Y290, which is bulkier than H290, is predicted to block the solvent exposure of the ε-NH_3_ group of the K320 residue more effectively than H290 (Fig. 5). In summary, significant energy stabilization due to Y290–K320 interaction also has the potential to physically protect K320 residue from ubiquitination.

**Fig. 8.**
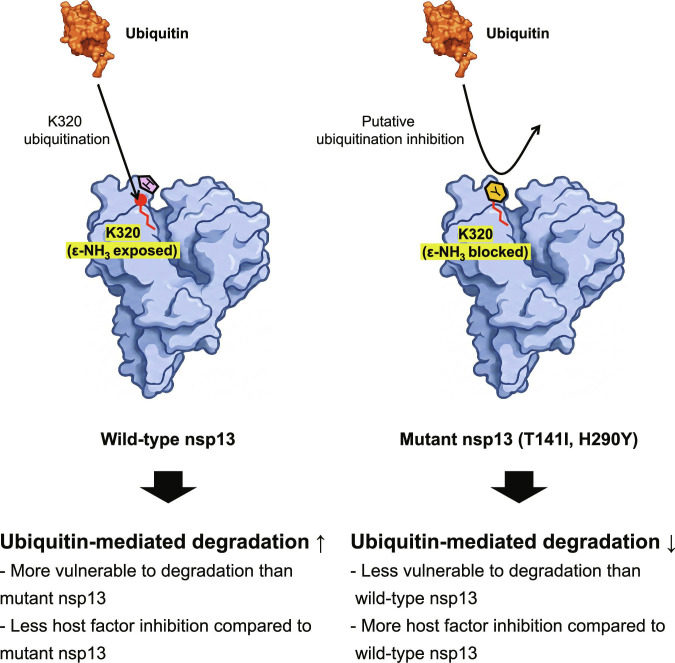
Proposed working model of the point mutations in nonstructural protein 13 (nsp13). The H290Y substitution is predicted to block the side chain of K320, thereby decreasing the ubiquitination of nsp13; this is because Y290 is bulkier than H290 and Y290–K320 interaction was calculated to have stronger attractive energy than H290–K320 interaction. This ubiquitination-resistant nsp13 mutant can block various host factors related to innate immunity more efficiently than the wild-type nsp13.

These results suggest that N15 nsp13 is potentially better protected from ubiquitin-induced degradation compared to wild-type nsp13. We propose the model that the H290Y mutation enhances nsp13 stability and its innate immune antagonistic ability to suppress viral sensing pathways (Fig. 8). Based on this model, N15 nsp13 can maintain optimal replicative fitness while maximizing its stealth duration within the host cell, suggesting a possible mechanism for decoupling viral progeny production from host immune detection.

However, shielding K320 by the H290Y substitution remains a computational prediction. Furthermore, while it remains to be functionally proven whether a larger SASA value of the lysine side chain’s ε-NH_3_ strictly leads to more ubiquitin conjugation, it is an assumption based on the general chemical and biological principle that a participating functional group (K320 ε-NH_3_) must be sterically exposed for a conjugation reaction to occur. Therefore, future experimental verification—such as cycloheximide chase experiments, targeted ubiquitination assays, and proteasome inhibition studies—are essential to definitively validate the effects of the H290Y mutation on nsp13 stability and turnover *in vivo*.

The INTAA results also imply that the H290Y substitution has the potential to affect the microenvironment of the ATP-binding site. Abnormally enhanced intramolecular locking between Y290 and K320 is predicted to structurally compete with or hinder the interaction of the corresponding residue with ATP. Changes in this interaction network could potentially affect the ATPase and RNA helicase kinetics of nsp13. However, in Fig. 2, there were no changes in replication kinetics in the N15 strain compared to those in other SARS-CoV-2 strains. Furthermore, as shown in Fig. S2, ATP-binding affinity does not differ significantly between the nsp13 wild type and mutant nsp13. This is because both H290 and Y290 can form π–π interactions with the adenine ring of ATP. Furthermore, structural studies on helicases have shown that the tyrosine residue in the π-stacking motif following the Walker A loop, typically found in ATP-binding cassette transporters, is replaced by H290 in SARS-CoV-2 helicase [69,70]. This suggests that histidine and tyrosine are compatible at residue 290 of nsp13.

Predicting the interaction between nsp13 and host factors was not performed because precise structural information regarding the interaction between nsp13 and host cell factors does not exist in the PDB. An existing *in silico* docking simulation (HDOCK) of nsp13 and TBK1 interaction shows that T141 and H290 of nsp13 are not the binding sites of TBK1 [71,72]. Furthermore, this analysis has the limitation that it was performed with a TBK1 monomer rather than a dimer. Its reliability is also limited considering reports that TBK1 interacts with the recA domain of nsp13 through its dimerization domain [21,64]. Therefore, actual experiments and structural information are required regarding the extent to which the nsp13 of the N15 strain inactivates host factors associated with viral infection.

Another protein variant that can induce a decrease in host cell cytokine-coding mRNA is the E protein. The E protein is a structural component of SARS-CoV-2 that functions as an ion channel responsive to the membrane lipid environment. It gates within the host cell via the N-terminal polar network extending from E8 to T9 to T11 in the endoplasmic-reticulum–Golgi intermediate compartment (ERGIC) system [73], thereby transporting calcium ions from the ERGIC lumen to the cytosol [22]. This calcium channel activity of the E protein activates the NLRP3 inflammasome, increasing the secretion of IL-1β and IL-18 [22,23]. In particular, T9I and T11A mutations found in omicrons are known to reduce tissue damage by decreasing the activity of the E protein [74,75], which suggests that T11M mutations will also lead to a loss of calcium ion channel activity.

The T11M substitution was predicted to induce structural instability of approximately 1.3 kcal/mol in the membrane environment in mCSM-membrane and MEMO-Stab2 results (Table 3). Since the 11th residue of the E protein is located near the membrane interface, the T11M substitution is expected to disrupt interactions with lipid head groups and the polar network composed of E8, T9, and T11 [40,73], adding credibility to the predictions of mCSM-membrane. In other words, from the perspective of the E protein monomer, the T11M mutation in the E protein is predicted to place it in a more unstable state than the wild type within a thermodynamic context that includes the membrane environment.

On the other hand, upon assembly, the T11M substitution is predicted to stabilize the pentameric structure and strengthen interchain interactions. As shown in Fig. 6, TMPfold analysis showed that the closed state of the T11M mutant has a lower $∆G_{\mathrm{asc}}$ than the closed state of the wild type. Also, regardless of the conformation change of the E protein, the magnitude of the L-J potential that acts on residue 11 was increased due to the T11M substitution through INTAA analysis (Fig. 7 and Fig. S4). The L-J potential gap between the closed state and the open state was also increased in the T11M pentamer.

The predictions from TMPfold and INTAA suggest that T11M substitution in the E protein is predicted to inhibit the transition between the closed and open states. The E protein is designed to switch between these 2 states in response to environmental changes to effectively transport calcium ions into the cytosol. However, the T11M mutation can disrupt this transition process. Specifically, in both the closed and open states, interactions between residue 11 and residues in adjacent chains are expected to be strengthened (Fig. S4). Particularly in the open state, residue 11 forms a strong hydrophobic packing with I13 of the adjacent chain (Fig. S4G and H). Considering that the conformation change of the E protein is induced by a uniaxial rotation of the α-helical transmembrane domain [40,73,76], the enhanced interchain interaction suggests that the T11M substitution has potential to inhibit the overall structural transition.

In summary, first, the T11M mutation in the E protein is predicted to induce instability in the monomer prior to pentameric assembly compared to the wild type. Second, the T11M substitution is predicted to hinder the smooth opening and closing of the channel even after pentameric assembly. This observation is analogous to the experimentally validated T11A mutation, which likewise replaces the polar threonine residue with a hydrophobic amino acid and has been shown to impair channel activity, cell lethality, and inflammatory cytokine release [74,75].

**Fig. 9.**
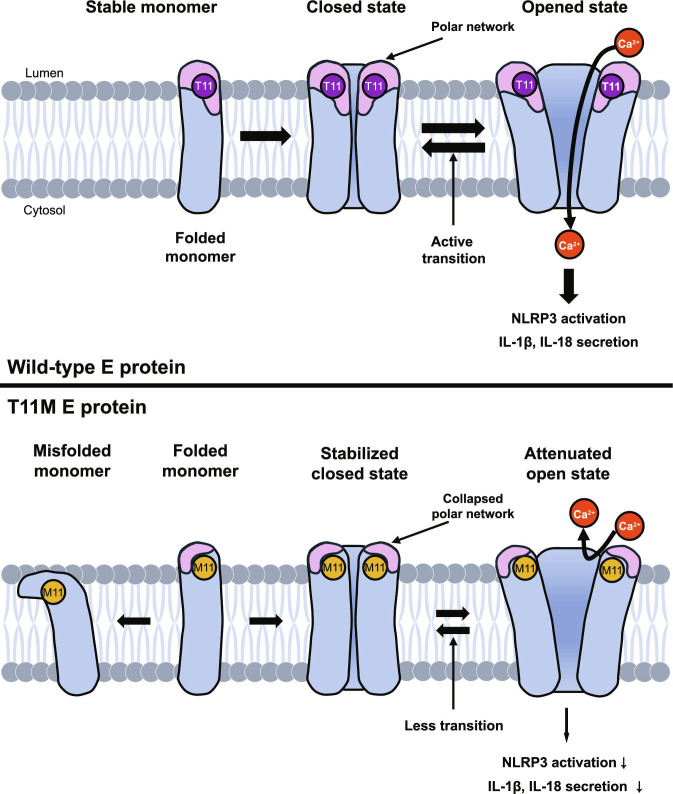
Proposed working model of the point mutation in the envelope (E) protein. The T11M substitution is predicted to destabilize the E protein monomer. However, this mutation stabilizes both the closed and open conformations of the E protein. Due to the stabilization of the pentameric assembly, the transition between the closed and open states is expected to be inhibited. The increased rigidity and the collapsed polar network could decrease NLRP3 activation, as well as the secretion of IL-1β and IL-18.

Therefore, we propose a working model illustrating how the T11M substitution contributes to the attenuation of NLRP3 inflammasome activation and subsequent decreases in IL-1β and IL-18 secretion, aligning with the observed attenuated phenotype (Fig. 9). According to this model, we hypothesize that the T11M substitution may decrease the calcium ion flux and reduce secretion of IL-1β and IL-18. This reduction could disrupt the inflammatory amplification loops, thereby suppressing the broad transcription of downstream inflammatory cytokines across the surrounding epithelium [77–79].

However, as no direct electrophysiological assessments or membrane-based molecular dynamics simulations were performed for the T11M variant in this study, our structural observations only provide predicted effects that are consistent with impaired viroporin function. Also, NLRP3 inflammasome activation due to calcium ion occurs at the posttranscriptional protein level (caspase-1 cleavage), meaning its inhibition cannot be directly confirmed using mRNA data alone. Therefore, further functional assays that test ion channel activity (molecular dynamics simulations and electrophysiological studies) and inflammasome activation (calcium flux measurements, caspase-1 cleavage analyses, and multiplex cytokine secretion assays) are essentially required to verify the proposed functional impairment of the E protein.

Our initial *in silico* analysis less highlighted potential functional impacts in several other mutated proteins such as nsp1, spike protein, and ORF3a protein. In addition, their topological and biological characteristics led us to deprioritize them as primary drivers of the observed N15 phenotype. The E91A substitution in nsp1 received high damage scores in PolyPhen-2 and PROVEAN; however, since this variant is known to have lost host mRNA degradation activity [59], it is difficult to regard it as the primary mechanism explaining the overall reduction of broad cytokine and antiviral protein mRNAs. In other words, this suggests that the N15 strain has adopted a more sophisticated immune evasion strategy than the simple immune evasion of total degradation of the host transcriptome.

Both the N709S and E1150D of the spike protein are located in the ectodomain [80], and given the orientation of the spike protein, direct interactions with host factors present in the cytosol are unlikely. Since N709 is a glycosylation site [81], N709S substitution could affect immune response or infection dynamics. However, current computational analyses are limited to evaluating changes in spike protein structure and ACE2 binding [13,15,16,18], and the effects of spike protein mutations on host immune responses and infection dynamics remain difficult to predict directly using *in silico* approaches alone.

The V259L mutation in ORF3a protein was found to have minimal functional impact in both predictive tools, and reliable *in silico* interaction analysis is limited due to its location in the intrinsically disordered region. Finally, although wild-type ORF8 is known to have immune evasion functions through MHC-I down-regulation [82,83], the L84S variant is a variant shared by the N15 and MA10 strains. Therefore, this mutation alone cannot explain the unique immunosuppressive phenotype of N15. Instead, the more severe immunosuppression observed in N15 is likely driven by the putative interplay of the nsp13 helicase and the E protein variants.

## Limitations and Future Directions

While our study examines N15 infection at the transcriptional level, the link between mRNA expression and actual protein secretion has not yet been experimentally confirmed. Future studies, including multiplex cytokine assays and enzyme-linked immunosorbent assay, are needed to determine whether these transcriptional changes lead to reduced immune signaling at the protein level in the host. Furthermore, it is worth noting that our experiments assessing viral kinetics and innate immune responses were conducted using a relatively high MOI with an MOI of 1.2. Although this high-inoculum approach was strategically selected to facilitate synchronized viral infection and minimize interreplicate variability across time points, it poses a structural limitation by potentially obscuring subtle, biologically relevant differences in host–pathogen dynamics. Because epithelial innate immune activation *in vivo* is deeply intertwined with the multicycle dynamics of viral spread and interferon propagation to neighboring cells, a high initial MOI might mask phenotypes that manifest only during lower inocula. Therefore, performing complementary experiments using lower MOIs such as an MOI of 0.01 or 0.1 in future functional validation studies will be essential to accurately dissect the viral spread kinetics and provide holistic insights into the stealth properties of the N15 isolate.

It is important to explicitly distinguish our experimentally demonstrated findings from our computationally inferred mechanisms. While our *in vitro* single-cell (Calu-3) models clearly demonstrate the unique virological phenotype of the N15 isolate by maintaining replication efficiency while significantly suppressing the host immune response, the underlying structural mechanisms proposed in this study are based entirely on computational predictions. We hypothesize that the immune-evasive phenotype of the N15 strain can be mediated by candidate determinants including the increased stability of nsp13 and the deterioration of the E protein’s ion channel function. Because the N15 genome contains multiple substitutions across various viral proteins, it remains unclear whether the observed phenotype is driven by these specific mutations individually, their synergistic effects, or interactions with other uncharacterized variants. Therefore, future reverse genetics approaches and experimental verification are required to confirm whether these putative mechanisms, such as altered nsp13 helicase kinetics, computationally inferred nsp13 stability via potential resistance to ubiquitination, and predicted impairment of the Ca^2+^ conduction of the E protein, actually lead to host immune suppression in *in vivo* infection models and how they might manifest in populations with varying degrees of reduced cellular or local immunity.

Extrapolating these computational and molecular-level findings to clinical cases involves caution due to the complex mixture of cellular receptors and the intricate interplay of local and systemic immunity. Furthermore, as this analysis was focused on the ancestral-like early-pandemic variant N15, additional research involving a broader panel of clinical isolates is necessary to determine whether these proposed mechanisms are conserved across other SARS-CoV-2 lineages. Nevertheless, this study provides a robust and systematic framework for identifying viral genetic determinants associated with attenuated immune evasion strategies. Our findings contribute to a deeper understanding of the molecular basis of differential viral pathogenesis, offering a foundational model for future investigations into the evolutionary trajectory of SARS-CoV-2–host interactions.

## Conclusion

This study characterizes an ancestral-like early-pandemic SARS-CoV-2 variant, N15, that combines efficient replication in human airway epithelial cells with markedly attenuated induction of cytokine and interferon responses. Despite genetic divergence from MA10, Beta, and Omicron, N15 displayed replication kinetics comparable to those of the other strains in Calu-3 cells, indicating that reduced innate immune activation occurs independently of viral growth efficiency. Through integrated genomic annotation and computer-based functional prediction, we identified a limited number of N15-specific amino acid substitutions and predicted that specific missense mutations in nsp1, nsp13, the spike protein, ORF8, and the envelope protein potentially impact these viral components. In particular, mutations in nsp13 and the envelope proteins were predicted to attenuate the overall host cell response. Taken together, these results suggest that changes in specific viral genetic characteristics and protein properties may enable the decoupling of efficient viral replication from the activation of innate immunity in epithelial cells. This work provides a foundation for prioritizing candidate viral determinants associated with attenuated host immune responses and serves as a framework for future experimental validation using well-defined viral and host models to elucidate the molecular basis of SARS-CoV-2–host interactions.

## Ethical Approval

The virus procedures were conducted at BSL3 in the Avison Biomedical Research Center, with the approval of the Institutional Biosafety Committee (IBC 2022-0320).

## Data Availability

All data generated or analyzed during this study are included in this published article and its Supplementary Materials.
