# Supplementary material for: Structural and Computational Insights into the Attenuated Innate Immune Recognition of the SARS-CoV-2 N15 Lineage, an Early-Pandemic Variant
Source: Comput Struct Biotechnol J. 2026 Aug 3;35(1):0175. doi: 10.34133/csbj.0175 (PMC13429915; doi:10.34133/csbj.0175)
Supplement: Supplementary 1 — Figs. S1 to S4 Tables S1 to S6 Movies S1 and S2 [file csbj.0175.f1.zip › Figure S4.pdf]

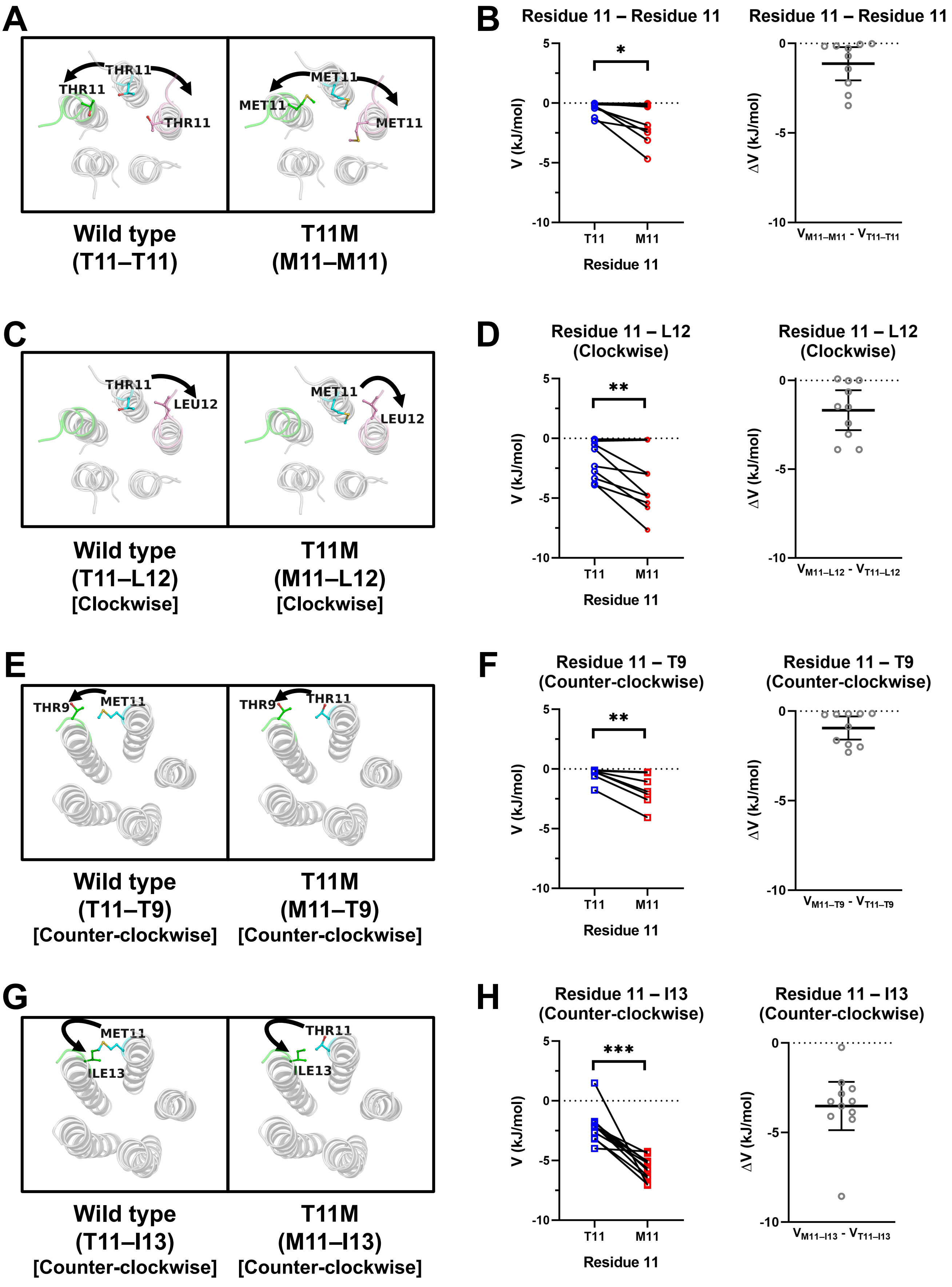
A
THR11
THR11
THR11
MET11
MET11
MET11
Wild type
(T11–T11)
T11M
(M11–M11)
B
Residue 11 – Residue 11
V (kJ/mol)
*
T11
M11
Residue 11
Residue 11 – Residue 11
ΔV (kJ/mol)
$V_{M11–M11}$ - $V_{T11–T11}$
C
THR11
LEU12
MET11
LEU12
Wild type
(T11–L12)
[Clockwise]
T11M
(M11–L12)
[Clockwise]
D
Residue 11 – L12
(Clockwise)
**
$V_{M11–L12}$ - $V_{T11–L12}$
E
THR9
MET11
THR9
THR11
Wild type
(T11–T9)
[Counter-clockwise]
T11M
(M11–T9)
[Counter-clockwise]
F
Residue 11 – T9
(Counter-clockwise)
**
$V_{M11–T9}$ - $V_{T11–T9}$
G
MET11
ILE13
THR11
ILE13
Wild type
(T11–I13)
[Counter-clockwise]
T11M
(M11–I13)
[Counter-clockwise]
H
Residue 11 – I13
(Counter-clockwise)
***
$V_{M11–I13}$ - $V_{T11–I13}$
