# Supplementary material for: Structural and Computational Insights into the Attenuated Innate Immune Recognition of the SARS-CoV-2 N15 Lineage, an Early-Pandemic Variant
Source: Comput Struct Biotechnol J. 2026 Aug 3;35(1):0175. doi: 10.34133/csbj.0175 (PMC13429915; doi:10.34133/csbj.0175)
Supplement: Supplementary 1 — Figs. S1 to S4 Tables S1 to S6 Movies S1 and S2 [file csbj.0175.f1.zip › sm.docx]

**
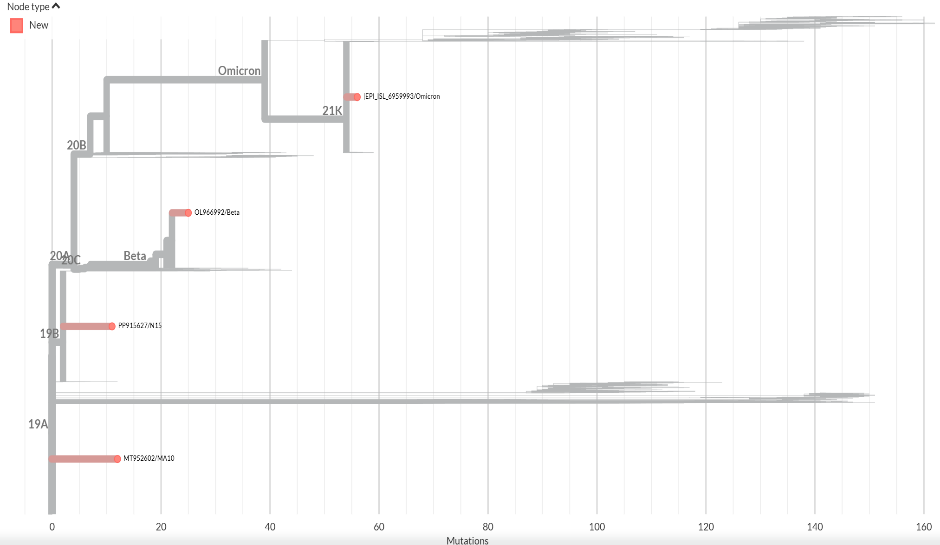

Figure S1. Global phylogenetic placement of SARS-CoV-2 strains analyzed in this study.** A global phylogenetic tree was constructed using the Nextclade platform based on complete genome sequences of representative SARS-CoV-2 strains, including N15 (GenBank accession no. PP195527), MA10 (MT952602), Beta variant (OL966992), and Omicron variant (EPI_ISL_695993). The Wuhan-Hu-1 reference genome (MN908947) was used as the ancestral reference for mutation calling and clade assignment. The x-axis indicates the number of nucleotide substitutions relative to the Wuhan-Hu-1 reference sequence, reflecting the cumulative genetic divergence of each strain. The y-axis represents the Nextstrain clade classification and their global distribution patterns. The strain analyzed in the present study is highlighted in red, allowing clear visualization of its phylogenetic relationship to globally circulating SARS-CoV-2 variants. This strain clusters within the corresponding Nextstrain clade (19A, 19B, Beta, or Omicron), demonstrating its genetic relatedness and evolutionary positioning among major SARS-CoV-2 lineages.

**Figure S2**. **Validation of the Boltz-2-predicted structures by comparison with the ATP-bound structure of nsp13 (PDB ID: 9I53) (A)** Predicted structures of the wild-type nsp13 and ATP. The structure shown in green represents PDB ID: 9I53, while other colors represent the predicted structures obtained from Boltz-2. **(B)** Predicted structures of the N15 nsp13 and ATP. The structure shown in green represents PDB ID: 9I53, while other colors represent the predicted structures obtained from Boltz-2. **(C)** root-mean-square deviations (RMSDs) between the 9I53 and the Boltz-2-predicted structures in (A) and (B). All Boltz-2-predicted structures show high similarity to the experimental ATP-bound structure of nsp13.


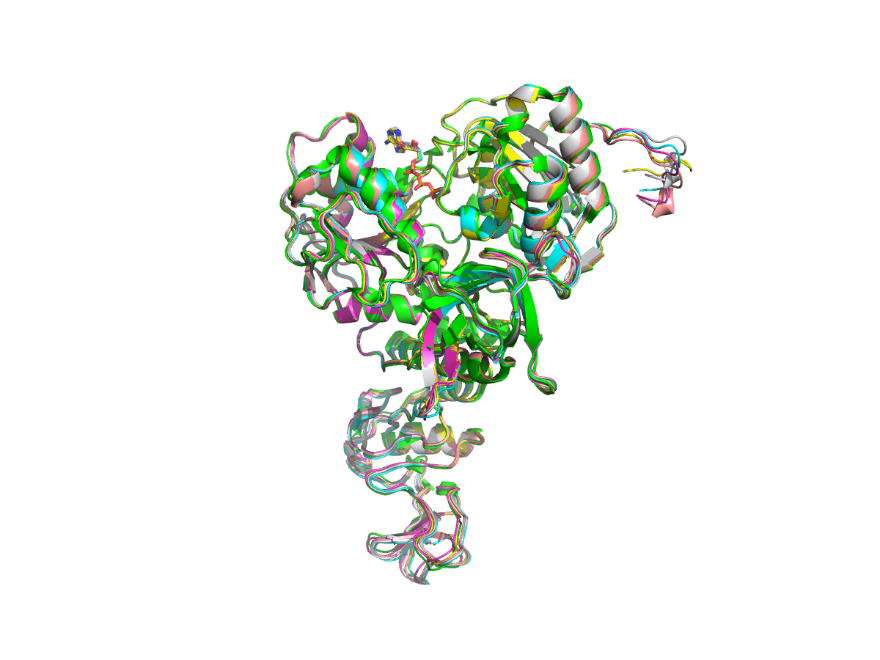

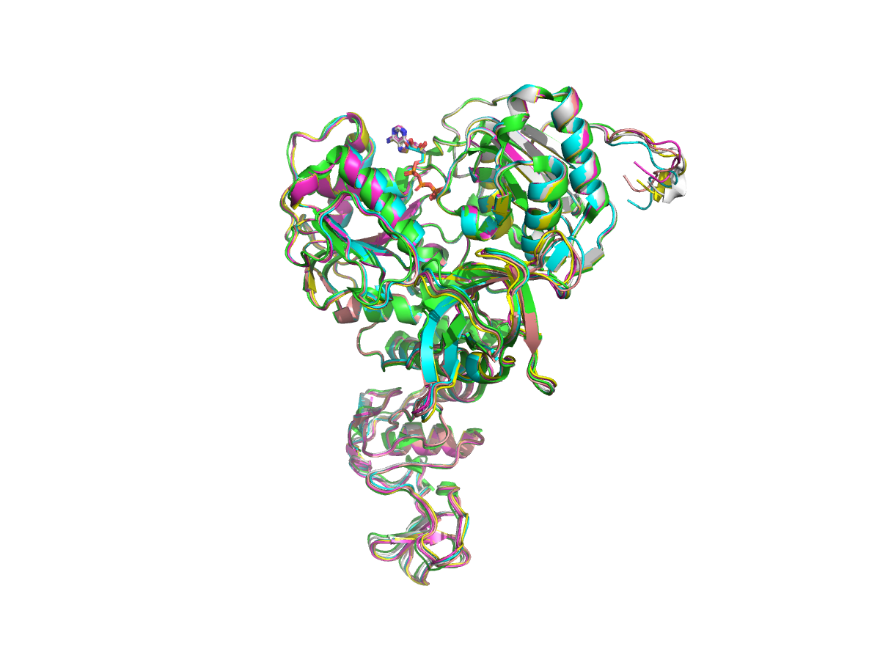


**A**

**B**

**C**

**Figure S3. Interaction energy profiles of residues 141 and 290 in nsp13 calculated by INTAA.** The PDB structure 7RDZ (apo-form of nsp13) resolves only 590 out of the total 601 amino acids in nsp13. **(A)** Interaction profile of T141 in the wild-type nsp13. **(B)** Interaction profile of I141 in the N15 nsp13. **(C)** Interaction profile of H290 in the wild-type nsp13. **(D)** Interaction profile of Y290 in the N15 nsp13. In (B), (C) and (D), the positions of L137 and K320 are indicated below the corresponding peaks. The ‘V’ on the y-axis of all graphs represents the summation of the Coulomb and L-J potentials. **(E)** Binding energy between residues 290 and K320, showing a stronger interaction in N15 (Y290) than in wild-type (H290).


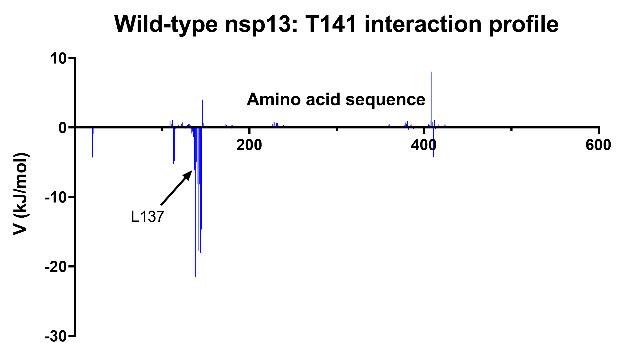

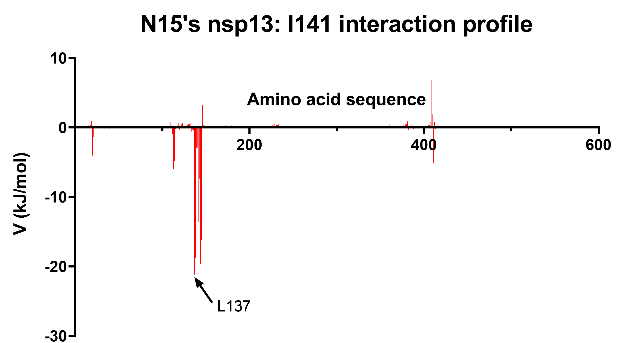

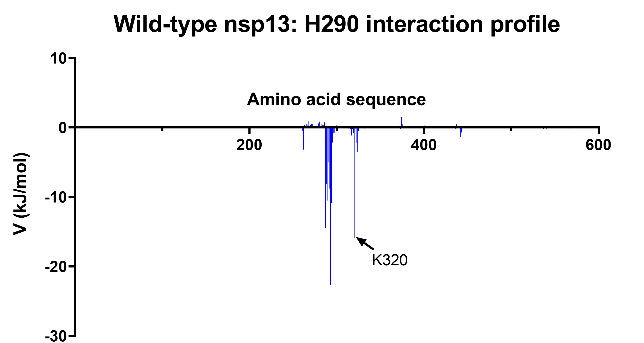

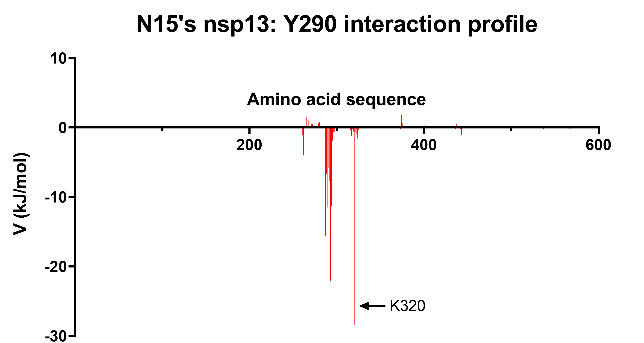

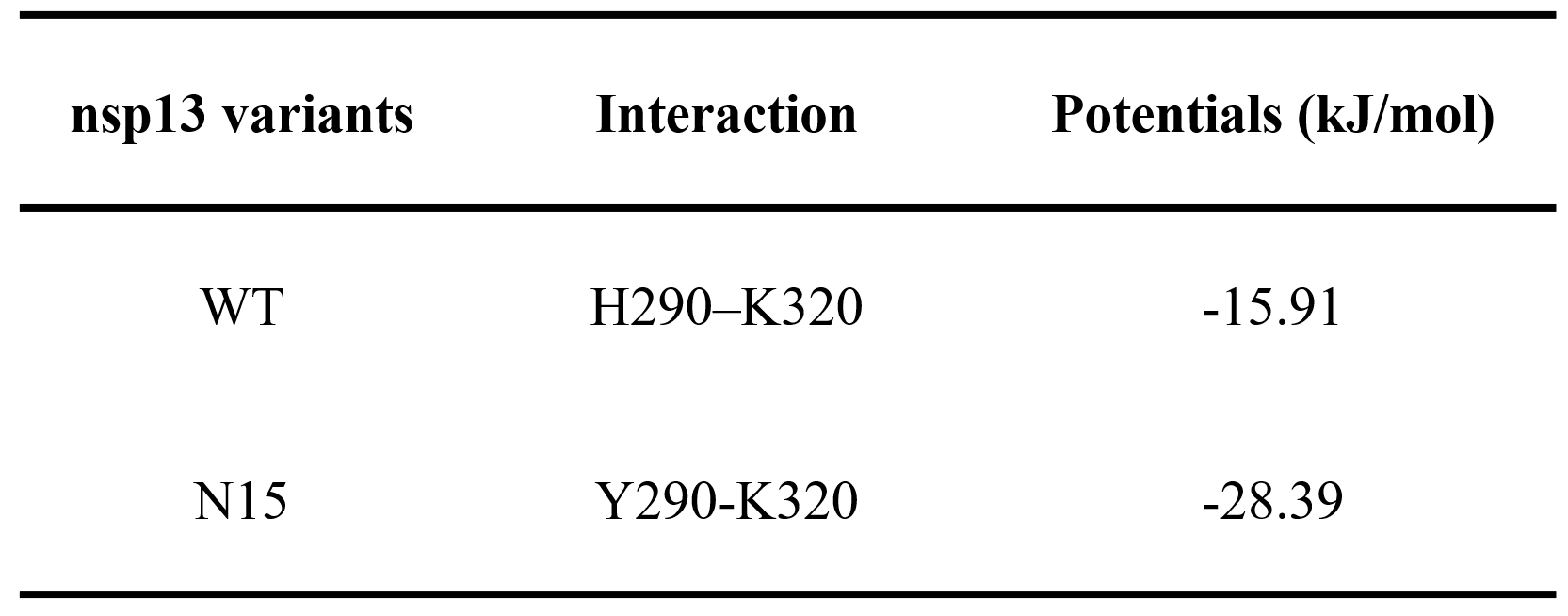


**A**

**B**

**C**

**E**

**D**

**
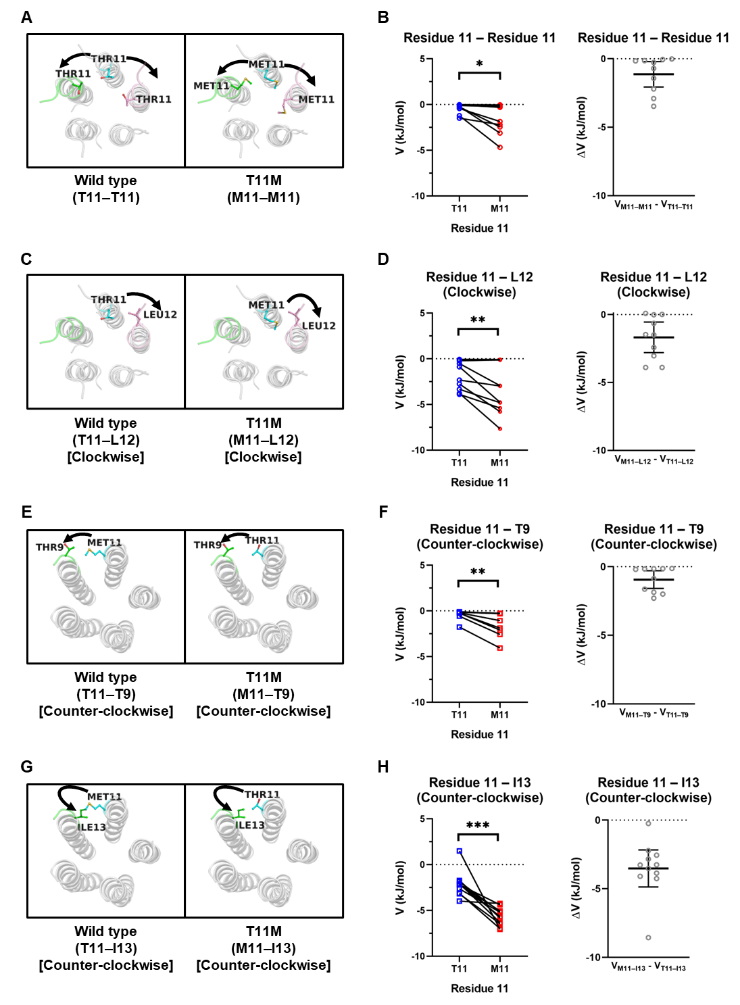
Figure S4.** Residues interacting with residue 11 and their L-J potentials in the wild-type and T11M E proteins. **(A)** Interactions of residue 11 with its counterpart in the adjacent chain of the closed E protein. **(B)** Comparison of L-J potentials between residues at position 11 for each variant (left panel) and the paired t-test difference plot (right panel). **(C)** Interactions of residue 11 with L12 in the clockwise adjacent chain of the closed E protein. **(D)** Comparison of L-J potentials between residue 11 and L12 (left panel) and the difference plot (right panel). **(E)** Interactions of residue 11 with T9 in the counter-clockwise adjacent chain of the open E protein. **(F)** Comparison of L-J potentials between residue 11 and T9 (left panel) and the difference plot (right panel). **(G)** Interactions of residue 11 with I13 in the counter-clockwise adjacent chain of the open E protein. **(H)** Comparison of L-J potentials between residue 11 and I13 (left panel) and the difference plot (right panel). Error bars represent the 95% confidence interval for the difference plots. The ‘V’ on the y-axis of all graphs represents the L-J potential.

**Table S1**. List of primers used in this study

| **Target Gene** | **Primer names** | **Sequence (5’-3’)** | **References** |
| --- | --- | --- | --- |
| Nucleocapsid | SARS-CoV2F-356 | CTGGACTTCCCTATGGTGCT | [1] |
|  | SARS-CoV2R-446 | CGGGTGCCAATGTGATCTTT |  |
|  | SARS-CoV2 Probe-394-415 | FAM-AGGCTCCCTCAGTTGCAACC-BHQ1 |  |
| β-ACTIN | Forward | TGGCATTGCCGACAGGATGC | [2] |
|  | Reverse | TCTGCTGGAGGTGGACAGCGA |  |
| IL1-β | Forward | AATCTGTACCTGTCCTGCGTGTT |  |
|  | Reverse | TGGGTAATTTTTGGGATCTACACTCT |  |
| IL-6 | Forward | ACCCCTGACCCAACCACAAAT |  |
|  | Reverse | AGCTGCGCAGAATGAGATGAGTT |  |
| IL-29 | Forward | GTGGTGCTGGTGACTTTGG |  |
|  | Reverse | CTCCTGTGGTGACAGAGATTTG |  |
| IFN-α | Forward | GTGAGGAAATACTTCCAAAGAATCAC |  |
|  | Reverse | TCTCATGATTTCTGCTCTGACAA |  |
| IFN-β | Forward | GCCGCATTGACCATGTATGAGA |  |
|  | Reverse | GAGATCTTCAGTTTCGGAGGTAAC |  |
| OAS1 | Forward | AGGTGGTAAAGGGTGGCTCC |  |
|  | Reverse | ACAACCAGGTCAGCGTCAGAT |  |
| human TNF-α | Forward | GCGTGGAGCTGAGAGATAAC | [3] |
|  | Reverse | TGAAGAGGACCTGGGAGTAG |  |
| IL-10 | Forward | GGCACCCAGTCTGAGAACAG | [4] |
|  | Reverse | ACTCTGCTGAAGGCATCTCG |  |
| ISG56 | Forward | TTGATGACGATGAAATGCCTGA | [5] |
|  | Reverse | CAGGTCACCAGACTCCTCAC |  |

[1] Chung HC, Kim SJ, Hwang SJ, Park SH, Park KM, Chung HW, et al. Isolation of a SARS-CoV-2 strain from pediatric patients in South Korea: biologic and genetic characterization. Frontiers in Microbiology 2025;16:1654224.

[2] Luangsay S, Gruffaz M, Isorce N, Testoni B, Michelet M, Faure-Dupuy S, et al. Early inhibition of hepatocyte innate responses by hepatitis B virus. Journal of Hepatology 2015;63:1314-1322.

[3] Li Y, Zhu Y, Feng S, Ishida Y, Chiu T-P, Saito T, et al. Macrophages activated by hepatitis B virus have distinct metabolic profiles and suppress the virus via IL-1β to downregulate PPARα and FOXO3. Cell Reports 2022;38.

[4] Li H, Zhai N, Wang Z, Song H, Yang Y, Cui A, et al. Regulatory NK cells mediated between immunosuppressive monocytes and dysfunctional T cells in chronic HBV infection. Gut 2018;67:2035-2044.

[5] Guo X, Chen D, Cai Q, Huang Z, Xu W, Peng L, et al. Minicircle DNA vector expressing interferon-lambda-3 inhibits hepatitis B virus replication and expression in hepatocyte-derived cell line. BMC Molecular and Cell Biology 2020;21:6.

**Table S2. PolyPhen-2-based in silico predictions of the functional impacts of amino acid substitutions in the SARS-CoV-2 N15 strain.** For each mutation, the affected protein, amino acid substitution, PolyPhen-2 model, score, qualitative prediction (benign, possibly damaging, or probably damaging), sensitivity, and specificity are listed.

| Gene/ORF | Mature peptide | Amino acid mutation^*^ | Model | PolyPhen-2 score | Prediction ^**^ | Sensitivity | Specificity |
| --- | --- | --- | --- | --- | --- | --- | --- |
| 1ab | leader protein (nsp1) | E91A | Human Divergence | **0.999** | **Probably damaging** | 0.14 | 0.99 |
|  |  |  | Human Variation | **0.998** | **Probably damaging** | 0.18 | 0.98 |
|  | Helicase (nsp13) | T141I | Human Divergence | **0.838** | **Possibly damaging** | 0.84 | 0.93 |
|  |  |  | Human Variation | 0.444 | Benign | 0.83 | 0.80 |
|  |  | H290Y | Human Divergence | **0.997** | **Probably damaging** | 0.41 | 0.98 |
|  |  |  | Human Variation | **0.994** | **Probably damaging** | 0.46 | 0.96 |
| S | Surface glycoprotein | N709S | Human Divergence | **0.963** | **Probably damaging** | 0.78 | 0.95 |
|  |  |  | Human Variation | **0.745** | **Possibly damaging** | 0.77 | 0.86 |
|  |  | E1150D | Human Divergence | 0.007 | Benign | 0.96 | 0.75 |
|  |  |  | Human Variation | 0.020 | Benign | 0.95 | 0.56 |
| 3a | ORF3a protein | V259L | Human Divergence | 0.017 | Benign | 0.95 | 0.80 |
|  |  |  | Human Variation | 0.006 | Benign | 0.97 | 0.45 |
| E | envelope protein | T11M | Human Divergence | **0.974** | **Possibly damaging** | 0.76 | 0.96 |
|  |  |  | Human Variation | **0.895** | **Possibly damaging** | 0.70 | 0.90 |
| 8 | ORF8 protein | L84S | Human Divergence | 0.061 | Benign | 0.94 | 0.84 |
|  |  |  | Human Variation | 0.009 | Benign | 0.96 | 0.49 |

^*^ Amino acid mutations are relative to the reference Wuhan-Hu-1 isolate (MN908947).

^**^PolyPhen-2 prediction categories: benign (0.0–0.5), possibly damaging (0.5–0.85), and probably damaging (0.85–1.0).

**Table S3. Detailed results of the PROVEAN-based *in silico* predictions.** For each mutation, the affected protein, PROVEAN score, qualitative prediction (deleterious or neutral), number of clusters, and number of supporting sequences used are listed. Mutations with a PROVEAN score of less than -2.5 are shown in **bold**.

| Protein | Mutation | PROVEAN score | Prediction | Number of clusters | Number of supporting sequences used |
| --- | --- | --- | --- | --- | --- |
| nsp1 | **E91A** | **-5.000** | **Deleterious** | 5 | 156 |
| nsp13 | **T141I** | **-3.389** | **Deleterious** | 30 | 421 |
|  | **H290Y** | **-5.300** | **Deleterious** | 30 | 421 |
| Spike glycoprotein | N709S | -0.363 | Neutral | 30 | 693 |
|  | E1150D | -0.660 | Neutral | 30 | 693 |
| ORF3a | V259L | -0.657 | Neutral | 3 | 37 |
| Envelope protein | **T11M** | **-3.067** | **Deleterious** | 5 | 25 |
| ORF8 | L84S | 2.333 | Neutral | 2 | 15 |

**Table S4. ESM-scan-based in silico prediction results and relative log-likelihood ratio (LLR) ranks within each protein.** For each mutation, the affected mature peptide, amino acid substitution, ESM-scan score, and within-protein LLR rank (percentile) are listed. Mutations with a percentile of less than 50 are shown in **bold**. A lower percentile (i.e., a lower LLR relative to all possible substitutions within that protein) indicates greater evolutionary constraint, suggesting that the mutation is less likely to occur.

| **Mature peptide** | **Amino acid mutation** | **ESM-scan (ESM2_650M*)** | **Within-protein LLR rank (Percentile)** |
| --- | --- | --- | --- |
| Leader protein (nsp1) | E91A | -0.11 | 60.85 |
| Helicase (nsp13) | T141I | -1.49 | 67.04 |
|  | H290Y | **-4.27** | **42.18** |
| Surface glycoprotein | **N709S** | **-2.00** | **33.06** |
|  | E1150D | -0.18 | 71.61 |
| ORF3a protein | V259L | -0.31 | 52.71 |
| Envelope protein | **T11M** | **-1.14** | **29.37** |
| ORF8 protein | L84S | 0.43 | 85.74 |

| **Rank** | **nsp1** |  | **nsp13** |  |  |  | **Spike** |  |  |  |
| --- | --- | --- | --- | --- | --- | --- | --- | --- | --- | --- |
|  | Mutation | LLR score | Mutation | LLR score | Mutation | LLR score | Mutation | LLR score | Mutation | LLR score |
| 1 | E91W | -2.35 | T141P | -3.46 | H290P | -11.70 | N709C | -7.49 | E1150C | -9.59 |
| 2 | E91M | -1.83 | T141W | -3.28 | H290D | -10.98 | N709W | -5.43 | E1150W | -6.86 |
| 3 | E91C | -1.62 | T141M | -2.23 | H290G | -10.10 | N709H | -4.40 | E1150H | -5.00 |
| 4 | E91Y | -1.38 | T141F | -2.13 | H290I | -9.74 | N709M | -4.38 | E1150M | -4.38 |
| 5 | E91N | -1.20 | T141C | -2.07 | H290K | -8.87 | N709R | -3.39 | E1150Y | -3.89 |
| 6 | E91I | -0.93 | T141L | -1.51 | H290E | -8.46 | N709Q | -3.11 | E1150R | -3.61 |
| 7 | E91K | -0.87 | ***T141I*** | -1.49 | H290A | -8.01 | N709Y | -3.05 | E1150F | -3.39 |
| 8 | E91H | -0.80 | T141G | -1.36 | H290V | -7.96 | N709K | -2.96 | E1150G | -2.40 |
| 9 | E91F | -0.78 | T141Y | -1.20 | H290L | -7.76 | N709F | -2.86 | E1150L | -2.25 |
| 10 | E91T | -0.77 | T141V | -0.93 | H290M | -7.36 | N709I | -2.74 | E1150P | -2.17 |
| 11 | E91Q | -0.72 | T141H | -0.59 | H290C | -6.79 | N709A | -2.44 | E1150I | -1.96 |
| 12 | E91P | -0.45 | T141S | -0.15 | H290N | -6.68 | N709E | -2.40 | E1150V | -1.84 |
| 13 | E91S | -0.39 | T141D | 0.01 | H290W | -6.39 | N709T | -2.36 | E1150N | -1.62 |
| 14 | E91R | -0.28 | T141A | 0.08 | H290S | -5.78 | N709L | -2.28 | E1150Q | -1.57 |
| 15 | E91D | -0.15 | T141N | 0.21 | H290R | -5.59 | N709P | -2.24 | E1150K | -1.51 |
| 16 | ***E91A*** | -0.11 | T141Q | 0.76 | H290F | -5.41 | N709V | -2.23 | E1150T | -1.44 |
| 17 | E91G | 0.06 | T141E | 0.83 | H290Q | -5.32 | N709D | -2.23 | E1150S | -1.05 |
| 18 | E91V | 0.24 | T141R | 1.07 | H290T | -4.94 | ***N709S*** | -2.00 | E1150A | -0.55 |
| 19 | E91L | 0.25 | T141K | 1.14 | ***H290Y*** | -4.27 | N709G | -1.97 | ***E1150D*** | -0.18 |

**Table S5. Log-likelihood ratio (LLR) rankings from ESM-scan comparing the mutations found in the N15 strain with all other possible substitutions at the corresponding positions.** For each evaluated position across the proteins, the table lists the rank, amino acid substitution, and LLR score. The specific mutations present in the N15 strain are highlighted in ***bold and italics***. All LLR values are color-coded using a red-white-blue spectrum based on their magnitude. The T11M mutation in the envelope (E) protein is specifically underlined.

**Table S5.** Continued

| **Rank** | **ORF3a** |  | **Envelope** |  | **ORF8** |  |
| --- | --- | --- | --- | --- | --- | --- |
|  | Mutation | LLR score | Mutation | LLR score | Mutation | LLR score |
| 1 | V259W | -3.54 | T11C | -1.57 | L84W | -1.97 |
| 2 | V259H | -1.99 | T11W | -1.39 | L84C | -1.51 |
| 3 | V259K | -1.84 | ***T11M*** | -1.14 | L84M | -1.51 |
| 4 | V259R | -1.83 | T11H | -0.93 | L84H | -0.65 |
| 5 | V259C | -1.67 | T11P | -0.66 | L84F | -0.42 |
| 6 | V259Q | -1.61 | T11Y | -0.60 | L84Y | -0.30 |
| 7 | V259F | -1.55 | T11G | -0.42 | L84R | -0.27 |
| 8 | V259Y | -1.39 | T11Q | -0.21 | L84I | -0.24 |
| 9 | V259E | -1.34 | T11F | -0.14 | L84A | -0.15 |
| 10 | V259D | -1.20 | T11N | -0.12 | L84Q | -0.09 |
| 11 | V259P | -1.18 | T11D | -0.11 | L84V | -0.07 |
| 12 | V259N | -0.92 | T11K | -0.10 | L84N | 0.06 |
| 13 | V259M | -0.70 | T11A | -0.07 | L84P | 0.14 |
| 14 | V259I | -0.43 | T11R | -0.06 | L84T | 0.14 |
| 15 | ***V259L*** | -0.31 | T11E | 0.00 | L84K | 0.16 |
| 16 | V259S | 0.03 | T11I | 0.06 | L84E | 0.32 |
| 17 | V259T | 0.10 | T11V | 0.18 | L84D | 0.36 |
| 18 | V259G | 0.30 | T11S | 0.39 | ***L84S*** | 0.43 |
| 19 | V259A | 1.29 | T11L | 0.46 | L84G | 0.52 |

**Table S6. MolProbity validation of the refined structures of the E protein pentamer.** The table presents the clash score, percentage of favored Ramachandran angles, percentage of favored rotamers, and overall MolProbity score for each of the 10 structural frames of the wild-type and T11M E protein variants (derived from PDB IDs 7K3G and 8SUZ).

| **Structure**  **(ID_variant)** | **Frame** | **Clash score** | **Ramachandran Favored**  **(%)** | **Rotamer Favored**  **(%)** | **MolProbity score** |
| --- | --- | --- | --- | --- | --- |
| 7K3G_WT | 1 | 3.14 | 96.55 | 100 | 1.33 |
| 7K3G_WT | 2 | 3.14 | 96.55 | 100 | 1.33 |
| 7K3G_WT | 3 | 1.57 | 96.55 | 100 | 1.13 |
| 7K3G_WT | 4 | 2.36 | 96.55 | 100 | 1.24 |
| 7K3G_WT | 5 | 1.96 | 100 | 96.3 | 0.96 |
| 7K3G_WT | 6 | 0.79 | 98.62 | 96.3 | 0.75 |
| 7K3G_WT | 7 | 2.36 | 95.86 | 100 | 1.3 |
| 7K3G_WT | 8 | 4.72 | 91.03 | 96.3 | 1.76 |
| 7K3G_WT | 9 | 1.18 | 100 | 96.3 | 0.83 |
| 7K3G_WT | 10 | 2.75 | 100 | 97.78 | 1.06 |
| 7K3G_T11M | 1 | 6.25 | 96.55 | 100 | 1.57 |
| 7K3G_T11M | 2 | 2.73 | 96.55 | 100 | 1.28 |
| 7K3G_T11M | 3 | 1.56 | 93.1 | 100 | 1.34 |
| 7K3G_T11M | 4 | 1.95 | 96.55 | 100 | 1.18 |
| 7K3G_T11M | 5 | 1.95 | 100 | 96.3 | 0.96 |
| 7K3G_T11M | 6 | 0.78 | 100 | 96.3 | 0.75 |
| 7K3G_T11M | 7 | 0.78 | 96.55 | 98.52 | 0.97 |
| 7K3G_T11M | 8 | 5.86 | 89.66 | 97.78 | 1.88 |
| 7K3G_T11M | 9 | 1.17 | 100 | 96.3 | 0.83 |
| 7K3G_T11M | 10 | 1.95 | 100 | 99.26 | 0.96 |

**Table S6**. Continued

| **Structure**  **(ID_variant)** | **Frame** | **Clash score** | **Ramachandran Favored**  **(%)** | **Rotamer Favored**  **(%)** | **MolProbity score** |
| --- | --- | --- | --- | --- | --- |
| 8SUZ_WT | 1 | 1.57 | 99.31 | 100 | 0.9 |
| 8SUZ_WT | 2 | 0.79 | 98.62 | 100 | 0.75 |
| 8SUZ_WT | 3 | 2.36 | 99.31 | 100 | 1.02 |
| 8SUZ_WT | 4 | 1.18 | 97.93 | 100 | 0.85 |
| 8SUZ_WT | 5 | 0.79 | 98.62 | 100 | 0.75 |
| 8SUZ_WT | 6 | 1.96 | 99.31 | 100 | 0.96 |
| 8SUZ_WT | 7 | 1.57 | 100 | 100 | 0.9 |
| 8SUZ_WT | 8 | 1.57 | 98.62 | 100 | 0.9 |
| 8SUZ_WT | 9 | 1.57 | 97.93 | 100 | 0.92 |
| 8SUZ_WT | 10 | 1.18 | 97.24 | 100 | 0.97 |
| 8SUZ_T11M | 1 | 1.17 | 98.62 | 100 | 0.83 |
| 8SUZ_T11M | 2 | 0.78 | 98.62 | 100 | 0.75 |
| 8SUZ_T11M | 3 | 1.56 | 100 | 100 | 0.9 |
| 8SUZ_T11M | 4 | 1.17 | 97.24 | 100 | 0.97 |
| 8SUZ_T11M | 5 | 0.78 | 99.31 | 100 | 0.75 |
| 8SUZ_T11M | 6 | 1.56 | 99.31 | 100 | 0.9 |
| 8SUZ_T11M | 7 | 0.78 | 99.31 | 100 | 0.75 |
| 8SUZ_T11M | 8 | 1.56 | 98.62 | 100 | 0.9 |
| 8SUZ_T11M | 9 | 1.56 | 98.62 | 100 | 0.9 |
| 8SUZ_T11M | 10 | 1.56 | 97.24 | 100 | 1.04 |
