# Supplementary figures and images for: Structural and Computational Insights into the Attenuated Innate Immune Recognition of the SARS-CoV-2 N15 Lineage, an Early-Pandemic Variant
Source: Comput Struct Biotechnol J. 2026 Aug 3;35(1):0175. doi: 10.34133/csbj.0175 (PMC13429915; doi:10.34133/csbj.0175)

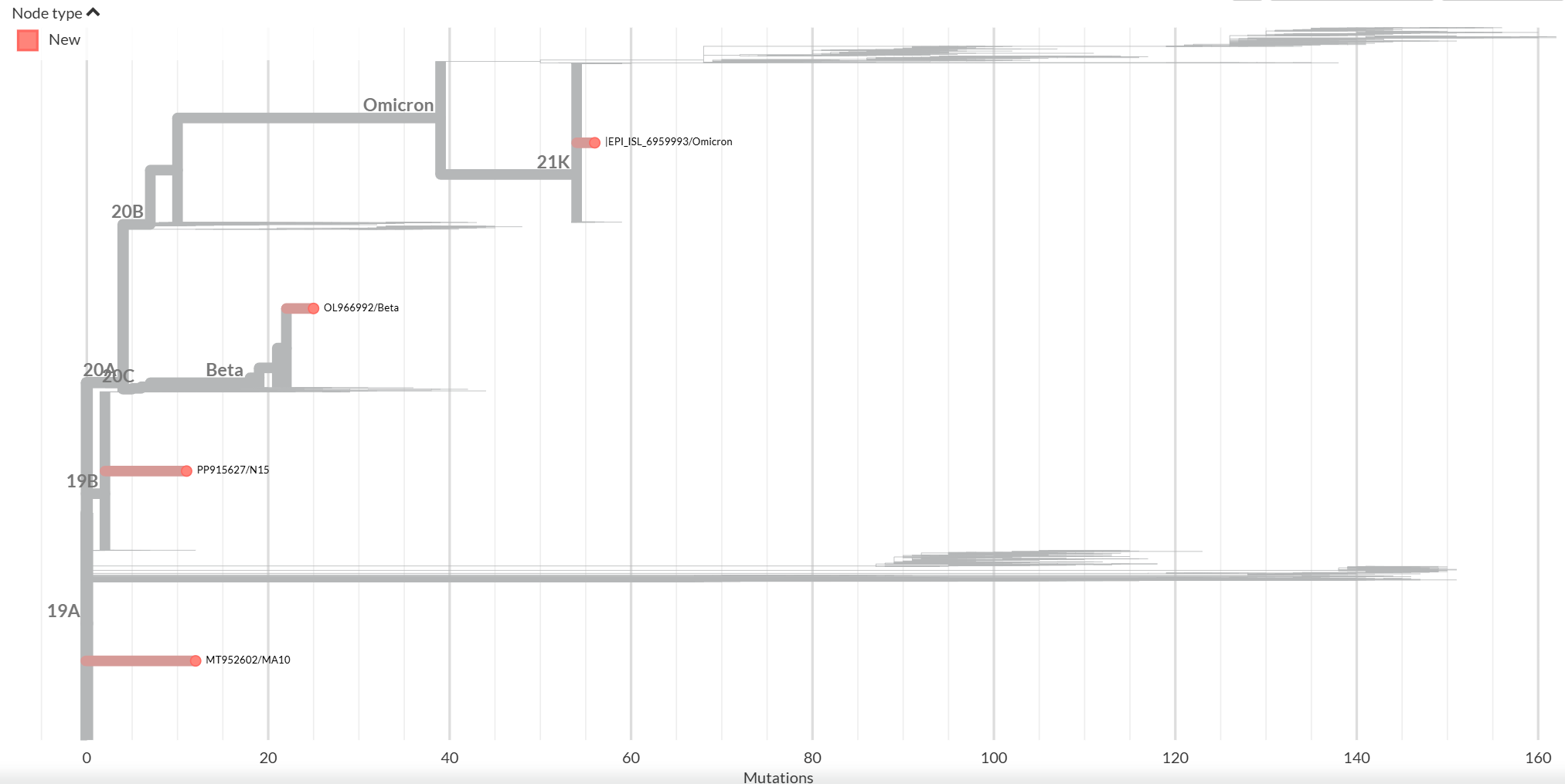

Supplement: Supplementary 1 — Figs. S1 to S4 Tables S1 to S6 Movies S1 and S2 [file csbj.0175.f1.zip › Figure S1.tif]

**A**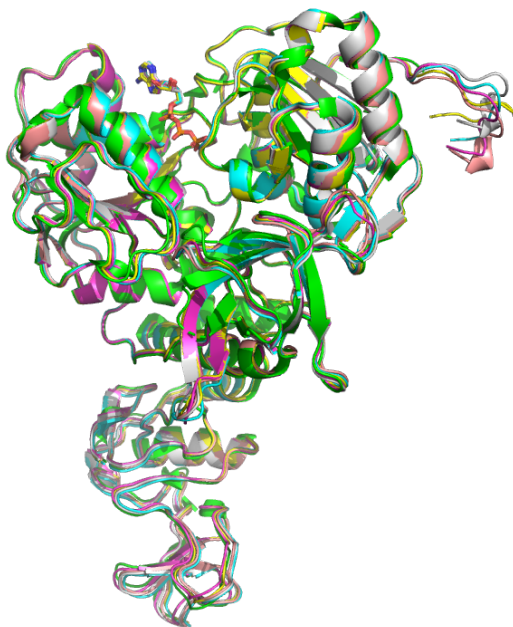**B**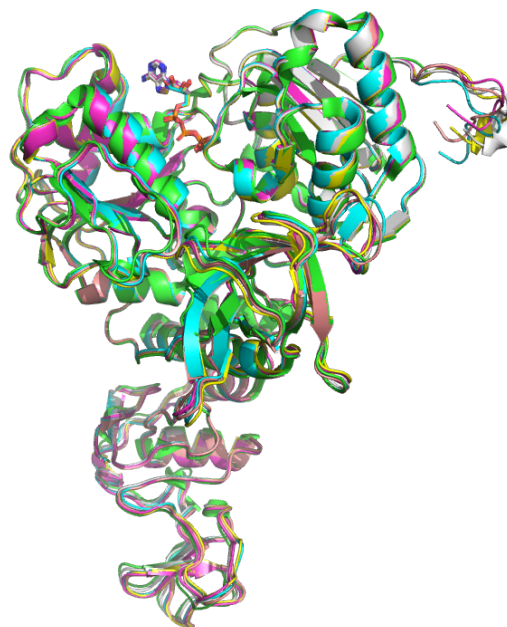**C**

| <b>RMSD compared to 9I53</b> | <b>WT</b> | <b>T141I, H290Y</b> |
|------------------------------|-----------|---------------------|
| Run 1                        | 0.824     | 0.630               |
| Run 2                        | 0.757     | 0.637               |
| Run 3                        | 0.576     | 0.678               |
| Run 4                        | 0.750     | 0.531               |
| Run 5                        | 0.912     | 0.698               |

Supplement: Supplementary 1 — Figs. S1 to S4 Tables S1 to S6 Movies S1 and S2 [file csbj.0175.f1.zip › Figure S2.pdf]

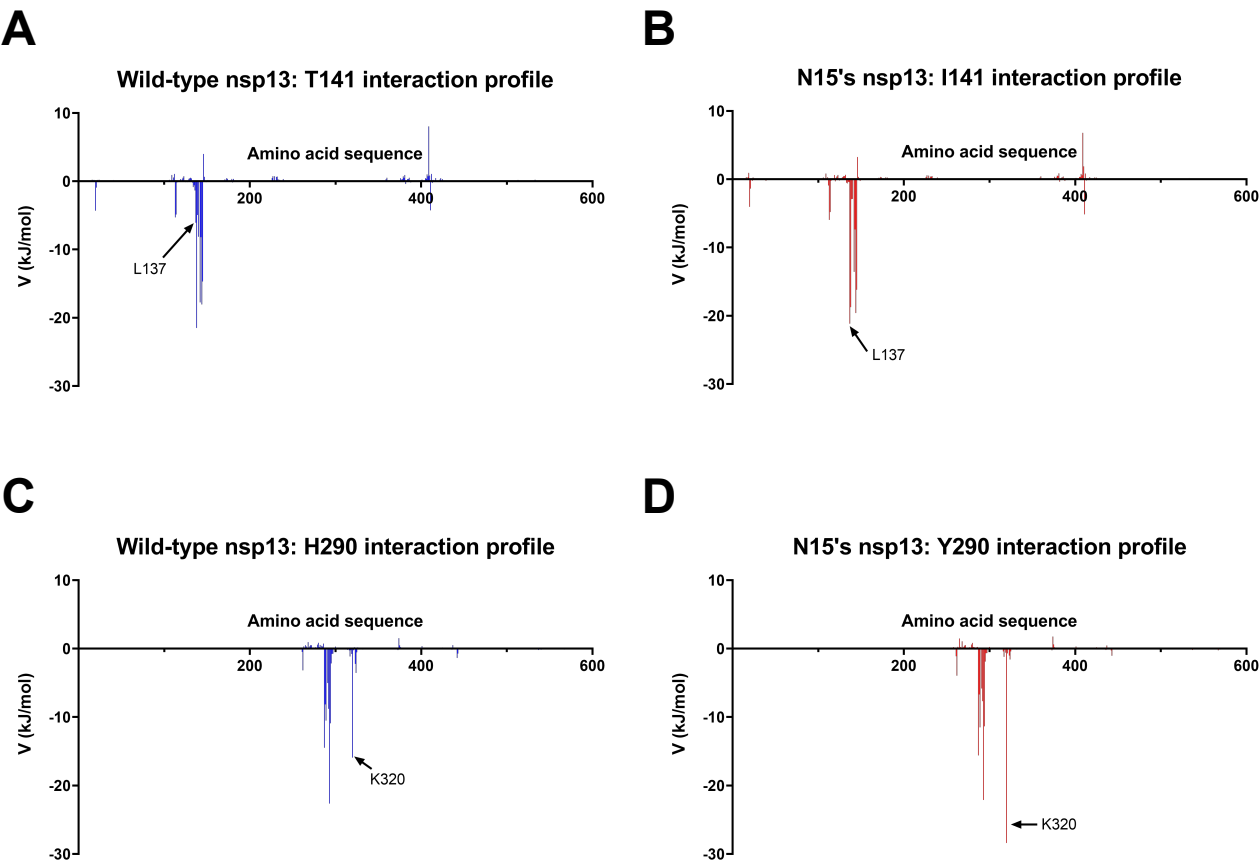

**E**

| nsp13 variants | Interaction | Potentials (kJ/mol) |
|----------------|-------------|---------------------|
| WT             | H290–K320   | -15.91              |
| N15            | Y290-K320   | -28.39              |

Supplement: Supplementary 1 — Figs. S1 to S4 Tables S1 to S6 Movies S1 and S2 [file csbj.0175.f1.zip › Figure S3.pdf]
